# Sni445 recruits box C/D snoRNPs snR4 and snR45 to guide ribosomal RNA acetylation by Kre33

**DOI:** 10.1093/nar/gkag030

**Published:** 2026-01-28

**Authors:** Jutta Hafner, Ingrid Zierler, Hussein Hamze, Sébastien Favre, Matthias Thoms, Natalia Kunowska, Sarah Rimser, Benjamin Albert, Tomas Caetano, Marion Aguirrebengoa, Roland Beckmann, Ulrich Stelzl, Dieter Kressler, Anthony K Henras, Brigitte Pertschy

**Affiliations:** Institute of Molecular Biosciences, University of Graz, 8010 Graz, Austria; BioTechMed-Graz, 8010 Graz, Austria; Institute of Molecular Biosciences, University of Graz, 8010 Graz, Austria; BioTechMed-Graz, 8010 Graz, Austria; Université de Toulouse, CNRS, UPS, 31062 Toulouse, France; Department of Biology, University of Fribourg, 1700Fribourg, Switzerland; Gene Center, University of Munich, D-81377Munich, Germany; Institute of Pharmaceutical Sciences, Pharmaceutical Chemistry, University of Graz, 8010 Graz, Austria; Institute of Molecular Biosciences, University of Graz, 8010 Graz, Austria; BioTechMed-Graz, 8010 Graz, Austria; Université de Toulouse, CNRS, UPS, 31062 Toulouse, France; Université de Toulouse, 31062 Toulouse, France; Université de Toulouse, 31062 Toulouse, France; Gene Center, University of Munich, D-81377Munich, Germany; BioTechMed-Graz, 8010 Graz, Austria; Institute of Pharmaceutical Sciences, Pharmaceutical Chemistry, University of Graz, 8010 Graz, Austria; Department of Biology, University of Fribourg, 1700Fribourg, Switzerland; Université de Toulouse, CNRS, UPS, 31062 Toulouse, France; Institute of Molecular Biosciences, University of Graz, 8010 Graz, Austria; BioTechMed-Graz, 8010 Graz, Austria

## Abstract

Eukaryotic ribosome synthesis is a highly complex, multistep process that is best characterized in the yeast *Saccharomyces cerevisiae*. It is orchestrated by >200 ribosome assembly factors and 75 small nucleolar ribonucleoproteins (snoRNPs), which guide site-specific chemical modifications of precursor rRNA (pre-rRNA). While canonical box C/D snoRNPs guide 2′-*O*-methylation, the atypical box C/D snoRNPs snR4 and snR45 guide acetylation of 18S rRNA residues C1280 and C1773, respectively, catalyzed by the acetyltransferase Kre33. Here, we identify and characterize Ynl050c/Sni445 as a novel ribosome assembly factor and previously unrecognized auxiliary component of the snR4 and snR45 box C/D snoRNPs. Sni445 associates with snR4 and snR45 in their free form and is required for their stable incorporation into 90S pre-ribosomes. Genetic interactions link Sni445 and the snR4 and snR45 snoRNAs to ribosomal proteins Rps20 (uS10) and Rps14 (uS11), which are positioned near the respective acetylation sites in the 40S subunit. Moreover, Sni445 physically interacts with Kre33 within the 90S pre-ribosome, and its absence abolishes acetylation of C1280 and C1773. Our findings suggest that Sni445 facilitates the recruitment of snR4 and snR45 snoRNPs to 90S particles and might promote their interaction with Kre33, thereby enabling the site-specific acetylation of 18S rRNA by Kre33.

## Graphical Abstract

**Graphical Abstract:**
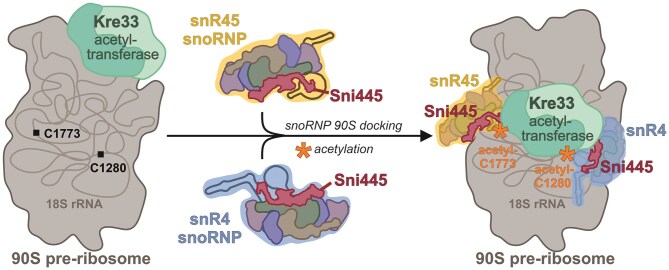

## Introduction

Ribosomes are essential macromolecular complexes that translate mRNA into proteins in all domains of life. In eukaryotes, ribosomes are composed of two subunits: the small 40S subunit and the large 60S subunit. The 40S ribosomal subunit contains the 18S rRNA and 33 ribosomal proteins (r-proteins). The 60S subunit consists of three rRNA species, 5S, 5.8S, and 25S rRNA in the model eukaryote *Saccharomyces cerevisiae* (yeast), and 5S, 5.8S, and 28S rRNA in humans, as well as 46 r-proteins in yeast and 47 in humans [1, 2]. The rRNA undergoes extensive modification, primarily through 2′-*O*-methylation and pseudouridylation. Other modification reactions include base methylation and acetylation. Most of the corresponding modifications are introduced during the early steps of ribosome biogenesis [3, 4].

Ribosome biogenesis is a conserved multistep process that has been best characterized in yeast. More than 200 assembly factors transiently associate with pre-ribosomal particles to facilitate their maturation. This process begins in the nucleolus, where RNA polymerase I synthesizes the 35S pre-rRNA, a precursor for the mature 18S, 5.8S, and 25S rRNAs. These are flanked by external transcribed spacers (5′ ETS and 3′ ETS) and separated by internal transcribed spacers (ITS1 and ITS2). Spacer elements are then successively removed through endo- and exonucleolytic processing events (Supplementary Fig. S1A). Already co-transcriptionally, the earliest r-proteins and assembly factors associate with the pre-rRNA to form the 90S pre-ribosome. Cleavage at site A_2_ within ITS1 separates precursors for the 40S and 60S subunits, which then undergo independent maturation pathways in the nucleolus and nucleoplasm, followed by nuclear export and final cytoplasmic maturation, culminating in the production of functional ribosomes (reviewed in [5–10]).

Most rRNA modifications are introduced by small nucleolar ribonucleoproteins (snoRNPs), each comprising a small nucleolar RNA (snoRNA) and a set of four core proteins, including an enzyme. Through base pairing with the rRNA, snoRNAs guide the snoRNPs to their target sites, where the catalytic subunit introduces the modification. Two major classes of snoRNPs exist: box C/D snoRNPs, which mediate 2′-*O*-methylation, and box H/ACA snoRNPs, which catalyze pseudouridylation. In yeast, box C/D snoRNPs include the core proteins Nop56, Nop58, and Snu13, and the methyltransferase Nop1.

Of the 46 box C/D snoRNAs expressed in yeast, 42 function as 2′-*O*-methylation guides. The remaining four, i.e. U3, snR190, snR4, and snR45, do not guide methylation. U3 and snR190 instead play structural roles in ribosome assembly, whereas snR4 and snR45 guide the *N*^4^-acetylation of cytidines C1280 and C1773 in 18S rRNA [3, 4, 11–14] (Supplementary Fig. S1B). These acetylation-guide snoRNAs are proposed to base-pair with rRNA sequences flanking the target sites, creating an unpaired bulge containing the cytidine that is modified (Supplementary Fig. S1B, II). The acetyl groups are not installed by the canonical box C/D snoRNP core proteins, but instead by the conserved acetyltransferase Kre33 (NAT10 in humans). Kre33 catalyzes *N*^4^-acetylcytidine formation in both rRNA and tRNA, and additionally contributes to 90S pre-ribosome maturation through a modification-independent structural function [11, 15–17].

Here, we introduce a novel ribosome assembly factor Sni445, functioning as an accessory protein component of the snR4 and snR45 acetylation guide snoRNPs. Sni445 binds to the free snoRNPs already during their synthesis and is required for their docking to 90S pre-ribosomal particles. There, Sni445 interacts with the acetyltransferase Kre33, probably stabilizing the contact between Kre33 and snR4/snR45, thereby facilitating 18S rRNA acetylation.

## Materials and methods

### Yeast strain and plasmid construction

All *S. cerevisiae* strains that were used in this study are listed in Supplementary Table S1 and were generated by using established methods for chromosomal deletion and tagging [18, 19]. To introduce the *kre33.R637A* allele into the chromosome, a knock-in strategy based on homologous recombination was employed. For this purpose, a plasmid was constructed containing a recombination cassette composed of: (i) the 3′-terminal 1800 nucleotides of either the *KRE33* wild type or *kre33.R637A* allele; (ii) 150 bp of downstream sequence corresponding to the *KRE33* terminator; (iii) a hygromycin resistance cassette; and (iv) chromosomal sequence spanning nucleotides 226–683 downstream of the *KRE33* open reading frame. The recombination cassette was PCR-amplified using the plasmid as a template, and the resulting linear PCR product was transformed into *RPS20* shuffle and *RPS14* shuffle strains. Hygromycin-resistant clones were selected on drug-containing plates, and correct genomic integration was confirmed by colony PCR and subsequent sequencing.

All plasmids generated in this study are listed in Supplementary Table S2 and were constructed using well-established molecular cloning methods employing restriction enzymes. All DNA fragments that were generated by PCR were confirmed by sequencing.

### Microscopy

Yeast cells expressing C-terminally green fluorescent protein (GFP)-tagged Sni445 and the nucleolar marker protein Nop58-RedStar2 from the genomic locus were examined under a fluorescence microscope in logarithmic growth phase. For the anchor away technique [20], yeast cells genomically expressing the Sni445-FRB-GFP [FKBP12-rapamycin-binding domain of human target of rapamycin (mTOR)-GFP] fusion protein were grown in logarithmic growth phase and were then incubated for up to 120 min with 1 µg ml^−1^ rapamycin in the absence or presence of 10 µg ml^−1^ cycloheximide. After various incubation times, cells were visualized under the fluorescence microscope and compared with untreated cells. Both microscopy experiments were performed using a Leica DM6 B microscope with GFP or RHOD ET filters, a × 100/1.4 Plan APO objective, a DFC 9000 GT camera, and the LasX software.

### Yeast two-hybrid assays

Yeast two-hybrid (Y2H) assays were performed to analyze protein–protein interactions. For this purpose, the Y2H reporter strain PJ69-4A [21] was transformed with two different plasmids, one plasmid encoding the bait protein fused N-terminally to the DNA-binding domain of the Gal4 transcription factor (abbreviated as BDN or G4BDN, with a c-myc tag and a *TRP1* marker used for selection) and the other plasmid encoding the prey protein fused C-terminally to the transcription activation domain of the Gal4 transcription factor (abbreviated as ADC or G4ADC, with a HA-tag and a *LEU2* marker used for selection). As controls, plasmids carrying just the DNA-binding domain of the Gal4 transcription factor (BDN-empty) or just the activation domain of the Gal4 transcription factor (ADC-empty) were used. After transformation, yeast cells were spotted in 10-fold serial dilutions starting with OD_600_ 0.75 on plates lacking tryptophan and leucine (SDC-Leu, -Trp, serving as control plates); plates lacking tryptophan, leucine, and histidine (SDC-Trp, -Leu, -His; growth on these plates shows activation of the *HIS3* reporter gene, indicating a weak protein–protein interaction), or plates lacking tryptophan, leucine, and adenine (SDC-Trp, -Leu, -Ade; growth on these plates shows activation of the *ADE2* reporter gene, indicating a strong protein–protein interaction). Plates were incubated for 3 days at 30°C.

### TurboID-based proximity labeling

The plasmid expressing C-terminally TurboID-tagged Sni445 under the control of the copper-inducible *CUP1* promoter was transformed into a wild-type yeast strain (YDK11-5A). The proximity labeling experiment, mass spectrometry (MS), data analysis, and graphical representation were carried out as previously described [22]. Raw data have been deposited to the ProteomeXchange Consortium via the PRIDE partner repository (https://www.ebi.ac.uk/pride/)[23] with the dataset identifier PXD067909. Additionally, a table containing the MaxQuant output data as well as the processed data is provided in Supplementary Table S3.

### Genetic interaction tests

To analyze genetic interactions, *SNI445, SNR4*, or *SNR45* were deleted in *RPS14* or *RPS20* shuffle strains. The *RPS20* gene is present as a single genomic copy; the *RPS20* shuffle strain contains a chromosomal deletion of *RPS20* and a *URA3* plasmid expressing wild-type *RPS20*. In contrast, *RPS14* is present as two paralogs in the yeast genome, *RPS14A* and *RPS14B*. The *RPS14* shuffle strain carries deletions of both genes and a *URA3* plasmid expressing wild-type *RPS14A*. Moreover, *SNR4* or *SNR45* were knocked out or the *kre33.R637A* allele was chromosomally integrated in *RPS14* or *RPS20* shuffle strains. The resulting strains were transformed with *LEU2* plasmids carrying either wild-type or different alleles of *RPS20* and *RPS14*. After transformation, cells were streaked on SDC plates (SDC+ all) containing 1 g l^−1^ 5-fluoroorotic acid (FOA; Thermo Scientific) to select for cells that lost the *URA3* plasmid carrying the respective wild-type gene. Subsequently, cells were spotted on SDC-Leu agar plates in 10-fold serial dilutions starting with OD_600_ 0.75. Plates were incubated for 2–3 days at 25, 30, and 37°C. Cells which were not able to grow on 5-FOA-containing agar (*rps14a.R136A sni445*∆, *rps14a.R136A* ∆s*nr45*, and *rps14a.R136A kre33.R637A*), and hence were not viable without the *URA3* plasmid, and respective controls were spotted on SDC-Leu plates and SDC plates containing 5-FOA in serial dilutions starting with OD_600_ 2, and incubated at 30°C for 3–6 days.

### Cross-linking and analysis of cDNA experiment

For cross-linking and analysis of cDNA (CRAC) experiments, a yeast strain expressing from the chromosomal locus a Sni445 fusion protein bearing a C-terminal HTP-tag [His6 tag–tobacco etch virus (TEV) protease cleavage site–protein A tag] and an untagged wild-type strain (negative control) were used. The CRAC experiment was performed as previously described ([24, 25]). The cDNA samples were sent for Illumina NextSeq2000 deep sequencing (EpiRNA-Seq facility, CNRS, Université de Lorraine, INSERM).

CRAC-seq data processing was performed using the pyCRAC toolkit (version 1.5.1) (https://sandergranneman.bio.ed.ac.uk/pycrac-software), a suite of Python-based tools specifically designed for the analysis of CRAC data. The analysis pipeline was implemented using Snakemake (version 8.5.5), ensuring workflow reproducibility, modularity, and scalability. Each step of the pipeline was encapsulated within dedicated conda environments, guaranteeing consistent software dependencies across executions.

- Raw reads were first demultiplexed using pyCRAC’s “pyBarcodeFilter.py” with one mismatch allowed.

- Raw reads were quality-checked using FastQC (v0.12.1) and aligned to the _Saccharomyces cerevisiae_ pyCRAC’s reference genome (Saccharomyces_cerevisiae.EF2.59.1.0.fa) using Novoalign (v3.09.00) with the “-r Random” parameter to randomly assign multimapping reads. Both “.sam” and “.novo” output files were retained to support downstream analyses: “.sam” files were used to generate counts per million (CPM)-normalized BigWig coverage tracks using “samtools” (v1.14) for sorting and indexing, and “deepTools” (v3.4.3) for coverage computation (“bamCoverage”); “.novo” files were used as input for pyCRAC’s internal tools requiring alignment metadata.

- The pyCRAC’s reference GTF annotation file (Saccharomyces_cerevisiae.EF2.59.1.3.gtf) was first checked using PyCRAC’s “pyCheckGTFfile.py”. Gene name information was extracted using PyCRAC’s “pyGetGeneNamesFromGTF.py”, and a tabular genome file required by pyCRAC was generated from the reference FASTA using a custom Python script.

- PyCRAC’s pyReadCounters.py was used to quantify the number of cross-linking events per transcript. The tool was run without the “-blocks” option, using various values for the “-readLength” parameter; in this study we generated data at 30 and 1000 bp read lengths. All hits were analyzed without limitation applied.

- Pileup profiles of cross-link events were generated using PyCRAC’s pypileup.py. This tool was run with default parameters with pyreadCounter output gtf as input.

All configuration files, custom scripts, and conda environment definitions used to run the workflow are available upon request, enabling full reproducibility of the analysis.

Next-generation sequence (NGS) analysis files of raw and processed data were deposited in the Gene Expression Omnibus database under the accession number GSE299803.

### Tandem affinity purification

Cells expressing chromosomally C-terminally tandem affinity purification (TAP)-tagged (calmodulin-binding peptide–TEV protease cleavage site–protein A tag) fusion proteins were used for TAP purifications. To this end, cells were grown in 2 liters of YPD to an OD_600_ of 1.8, harvested, and frozen at -20°C. Pellets were suspended in lysis buffer [50 mM Tris-HCl pH 7.5, 100 mM NaCl, 1.5 mM MgCl_2_, 0.075% NP-40, 1 mM dithiothreitol (DTT, Roth), and 1× Protease Inhibitor Mix FY (Serva)] and lysed by mechanical disruption using glass beads. After a centrifugation step, the cleared cell lysates were incubated with 300 µl of IgG Sepharose™ 6 Fast Flow (GE Healthcare) for 1.5 h at 4°C. IgG beads were then transferred into Mobicol columns (MoBiTec) and washed with 10 ml of lysis buffer. After washing the beads, 300 µl of buffer containing TEV protease was added and bound proteins were eluted for 70 min at room temperature. CaCl_2_ was added to TEV eluates to a final concentration of 2 mM, and the TEV eluate was then incubated with 65 µl of Calmodulin Sepharose™ 4B (GE Healthcare) for 1 h at 4°C. After washing beads with 5 ml of buffer containing 2 mM CaCl_2_, elution of bound proteins was performed using 40 µl of elution buffer containing 5 mM EGTA for 20 min at room temperature. Eluates were then used for RNA isolation or samples were subjected to MS proteomics analysis. Proteins were additionally further analyzed on NuPAGE™ 4–12% Bis-Tris gels followed by staining with the NOVEX^®^ Colloidal Blue Staining Kit (Thermo Fisher).

### FLAG purification

Cells expressing C-terminally FLAG-tagged Sni445 from the genomic locus were grown in 2 liters of YPD to an OD_600_ of 1.8. Cells were harvested and frozen at -20°C. Pellets were suspended in lysis buffer (50 mM Tris-HCl pH 7.5, 100 mM NaCl, 1.5 mM MgCl_2_, 0.075% NP-40, 1 mM DTT, and 1× Protease Inhibitor Mix FY). Cells were then lysed by mechanical disruption using glass beads. After a centrifugation step, the cleared lysates were incubated with 300 µl of Anti-FLAG^®^ M2 Affinity Gel (Sigma Aldrich) for 1 h at 4°C. Beads were then transferred to Mobicol columns (MoBiTec) and washed with 10 ml of buffer. Elution of bound proteins was performed using buffer containing 100 µg ml^−1^ FLAG-peptide (Sigma Aldrich) for 1 h at 4°C. Eluates were then used for RNA isolation or samples were analyzed by MS analysis. Final samples were further analyzed on NuPAGE™ 4–12% Bis-Tris gels followed by staining with the NOVEX^®^ Colloidal Blue Staining Kit (Thermo Fisher).

### Split tag purification of Sni445-TAP and Nop58-FLAG

Cells were grown to an OD_600_ of ∼3, harvested by centrifugation, flash-frozen in liquid nitrogen, and stored at -80°C. Cells were lysed using a SPEX 6970EFM Freezer/Mill. Cell powder was resuspended in buffer containing 60 mM Tris-HCl pH 7.5, 50 mM NaCl, 40 mM KCl, 5 mM MgCl_2_, 1 mM DTT supplemented with 5% glycerol, 0.1% NP-40, and 1× EDTA-free protease inhibitor (Roche). The lysate was cleared by two successive centrifugation steps: first at 4 000 rpm for 15 min (Eppendorf 5810 R), and second at 17 500 rpm for 25 min (Sorvall LYNX 6000). Both steps were carried out at 4°C. The supernatant was incubated with pre-equilibrated IgG Sepharose™ 6 Fast Flow affinity resin and incubated at 4°C on a turning wheel for 90 min. IgG beads were collected by centrifugation and, after a batch wash, were transferred to a Mobicol column (MoBiTec) and washed with an additional 10 ml of lysis buffer by gravity flow. Bound proteins were eluted from the beads by the addition of homemade TEV protease by incubating for 90 min at 16°C. Anti-FLAG M2 agarose beads (Sigma-Aldrich) were added to the eluate and incubated for 60 min at 4°C. The beads were washed with buffer supplemented with 0.01% NP-40 and 2% glycerol and transferred to a Mobicol column (MoBiTec) for a final wash with 10 ml of buffer containing 0.05% Nikkol and 2% glycerol. Samples were eluted by the addition of 3× Flag peptide (Sigma-Aldrich, final concentration 250 µg ml^−1^) for 1 h at 4°C, and analyzed on a 4–12% polyacrylamide gel (NuPAGE, Invitrogen) stained with colloidal Coomassie. Co-purifying proteins were analysed by MS.

### Mass spectrometry proteomics

#### Sample processing

Purifications for MS proteomics were performed in three independent replicates. Eluates of the Sni445-FLAG and Sni445-TAP purification, as well as the Pwp2-TAP and Enp1-TAP purifications, were processed using S-Trap™ micro columns (ProtiFi, Cat# C02-micro-80) following the high recovery protocol recommended by the manufacturer and using trypsin (Pierce, Cat# 90 059) as the carrier protein. After elution from the columns, the samples were lyophilized and resuspended in 12 μl of 0.1% formic acid; then 1 μl thereof was used per MS injection.

#### Mass spectrometry

Tryptic peptide samples were analyzed on an UltiMate 3000 RSLC (Thermo Scientific) coupled to a TimsTOF PRO (Bruker) mass spectrometer. Peptides were separated on a reversed-phase C18 Aurora column (25 cm × 75 µm) with an integrated Captive Spray Emitter (IonOpticks) at a column temperature of 50°C and a flow rate of 300 nl min^−1^. Mobile phases were A, 0.1% (v/v) formic acid in water; and B, 0.1% (v/v) formic acid in acetonitrile. Fraction B was linearly increased from 2% to 25% in a 90 min gradient, then increased to 40% for 10 min, and finally further increased to 80% for 10 min, followed by re-equilibration. The column was washed between runs with 50% buffer B for 3 min, 80% buffer B for 3 min, and re-equilibration with 2% buffer B for 5 min. The spectra were recorded in data-independent acquisition (DIA) mode.

#### Data processing

The DIA data were quantified with DIA-NN v.1.9 using a synthetic fasta library computed from the UniProt *S. cerevisiae* protein database file (reviewed; March 20, 2022), with default settings. MS2 and MS1 mass accuracies were set to 20 ppm and scan window size was set to 9. Output was filtered at 0.01 false discovery rate (FDR).

#### Data analysis

The label-free quantification (LFQ) output table generated in DIA-NN was filtered, and only the proteins detected in at least three out of six MS runs for any of the pulldowns were kept. The LFQ values were then log_2_-transformed, *z*-score- and bait-normalized, and the values for each biological replicate were averaged across the two MS runs. Next, the mean value for the three biological replicates was calculated and the missing values were imputed using Perseus style imputation with random values drawn from a normal distribution downshifted by 1.8 standard deviation (SD) with a width of 0.3 for each sample. Finally, the difference between averaged log_2_ intensity values of Sni445-TAP and Sni445-FLAG, Pwp2-TAP and Pwp2-TAP *sni445*Δ, and Enp1-TAP and Enp1-TAP *sni445*Δ, and the average normalized LFQ from all replicates were calculated.

The MS proteomics raw data together with the processing files were deposited to the ProteomeXchange Consortium using the PRIDE partner repository (https://www.ebi.ac.uk/pride/) with the dataset identifier PXD065447.

### Northern blotting

For RNA isolation, crude extract and final eluate from TAP or FLAG purifications were adjusted to a final volume of 600 µl with the respective buffers. RNA was extracted in two rounds of phenol-chloroform-isoamyl alcohol (25:24:1) extraction followed by one round of chloroform-isoamyl alcohol (24:1) extraction. RNA was then precipitated by adding 1/10 volume of 3 M sodium acetate pH 5.2, 2.5 volumes of 100% ethanol, and 1 µl of GylcoBlue™ (Invitrogen). After precipitation overnight at -20°C and pelleting of precipitated RNA by centrifugation, RNA pellets were dissolved in nuclease-free water. For analysis of snoRNAs, RNA samples were then loaded on NuPAGE™ 6% TBE-Urea gels (Thermo Fisher) followed by blotting onto Hybond N^+^ nylon membranes (Amersham Biosciences) using 0.5× TBE buffer at 300 mA.

For analysis of (pre-)rRNAs, 3 µg of RNA per lane were separated on 1.6% MOPS-agarose gels containing 20 mM 3-(*N*-morpholino)-propanesulfonic acid (MOPS), 5 mM sodium acetate, 1  mM EDTA, 0.75% formaldehyde, and ethidium bromide (pH 7.0), transferred overnight onto Hybond N^+^ nylon membranes (Amersham Biosciences) by capillary transfer, and UV cross-linked to the membrane.

To detect different snoRNA or (pre-)rRNA species, oligonucleotides complementary to the snoRNAs of interest were 5′-^32^P-labeled using T4 polynucleotide kinase and γ-[^32^P]ATP [185 TBq (5000 Ci) mmol^−1^ (Hartmann analytics)]. To enhance signals, several oligonucleotide probes complementary to different regions of the same snoRNAs were combined. The following oligonucleotides were used: snR4-1, 5′-CATCGACCCAGGAAAGCATCTTACAC-3′; snR4-2, 5′-CTAGAGTTATTTTAAAACAC-3′; snR4-3, 5′-CTATAACCTATCCTCATCGACTG-3′; snR45-1, 5′-CAGATCGCTCCGAGAAGAATTG-3′; snR45-2, 5′-GCGCAGGAACCGCTATCTCC-3′; snR45-3, 5′-CATTCTTAAGAATGTAACAAGATCAATGGG-3′; U3, 5′-GGATTGCGGACCAAGCTAA-3′; snR10-1, 5′-CCTTGTCGTCATGGTCGAATCG-3′; snR10-2, 5′-TCCTTGCAACGGTCCTCATC-3′; snR35-1, 5′-GAAGCCTAAACTTCCCTCAATTTCCTACAC-3′; snR35-2, 5′-CCAAAAGAGACTCGATATAAACAACACGG-3′; snR35-3, 5′-GCATGTCTGTCCTACCAGCCCTTGCATAGGCG-3′; U14-1, 5′-GGAACCAGTCTTTCATCACCGTG-3′; U14-2, 5′-CTCAGACATCCTAGGAAGGTCTCTAAAGAAGAGCGG-3′, U14-3, 5′-GTTAAGGAACCAGTCTTTCATCACCGTG-3′; snR77-1, 5′-GTTTTTGTTATAATCATCATATG-3′; snR77-2, 5′-CAACATATACTCGTTCAGCCAG-3′; snR77-3, 5′-CTAGGTCAGGATAGTGCAAAAACG-3′; D/A_2_ (20S), 5′-GACTCTCCATCTCTTGTCTTCTTG-3′; A_2_A_3_ (27SA2, 23S), 5′-TGTTACCTCTGGGCCC-3′; E/C2 (27S A + B), 5′-GGCCAGCAATTTCAAGTTA-3′; 18S, 5′-GCATGGCTTAATCTTTGAGAC-3′; and 25S, 5′-CTCCGCTTATTGATATGC-3′. Hybridization with the radiolabeled oligonucleotides was performed at 37°C overnight in buffer containing 0.5 M Na_2_HPO_4_, pH 7.2, 7% sodium dodecylsulfate (SDS), and 1 mM EDTA. After three subsequent washing steps with a buffer containing 40 mM Na_2_HPO_4_, pH 7.2, 1% SDS, signals were detected by exposing X-ray films. Membranes were regenerated by washing in 1% SDS prior to hybridization. X-ray films were scanned using a ChemiDoc Imager (Biorad), and band intensities were quantified using the Image Lab™ software (Biorad).

### Reverse transcription and misincorporation analysis of *N*^4^-acetylcytidine in 18S RNA

For detection of *N*_4_-acetylcytidines (ac4Cs), we adapted the assay developed in [26]. Cells were grown in 20 ml of YPD at 30°C to an OD_600_ of 0.7–0.9, and 4 OD_600_ units were harvested. Cells were resuspended in 200 µl of lysis buffer containing 40 mM Tris–HCl pH 7.5, 40 mM EDTA, and 2% SDS, and mechanically lysed by vigorous shaking with 200 µl of glass beads (0.5 mm diameter) for 3 min. RNA was extracted from the lysate by three extraction steps with phenol–chloroform–isoamyl alcohol (25:24:1) and one extraction step with chloroform–isoamyl alcohol (24:1). RNA was then precipitated by addition of 1/10 volume of 3 M sodium acetate (pH 5.2), 2.5 volumes of 100% ethanol, and 1 µl of GlycoBlue™ (Invitrogen), and, after drying, RNA was dissolved in 30 µl of nuclease-free water.

For chemical reduction of ac4C in 18S rRNA, 1 µg of the isolated RNA was incubated with either H_2_O or 100 mM sodium borohydride (NaBH_4_) in a final reaction volume of 100 µl. Samples were incubated at 37°C for 1 h, quenched with 15 µl of HCl (1 M), and neutralized by addition of 15 µl of Tris–HCl buffer (1 M, pH 8.0) in a final volume of 200 µl. RNA precipitation was performed by addition of 1/10 volume of 3 M sodium acetate (pH 5.2), 2.5 volumes of 100% ethanol, followed by centrifugation at 4°C, 15 000 rpm for 15 min. The RNA pellet was washed with 70% ethanol and, after drying, dissolved in 10 µl of nuclease-free water.

For reverse transcription, 200 pg of RNA from individual treatment reactions were incubated with 4 pmol of the respective primers [18S_h34_rev (5′-GGTTAAGGTCTCGTTCGTTATCG-3′) for analysis of the acetylated cytidine in helix h34, or 18S_h45_rev (5′-TAATGATCCTTCCGCAGGTTCACCTAC-3′) for analysis of the acetylated cytidine in helix h45], 10 nmol dNTP mix (NEB), and nuclease-free water in a final reaction volume of 10 µl, incubated at 65°C for 5 min, and promptly transferred on ice. Then 1× Induro RT Reaction Buffer (NEB), 8 U of murine RNase inhibitor (NEB), and 200 U of Induro Reverse Transcriptase (NEB) were added in a final volume of 20 µl. Samples were incubated at 55°C for 10 min, followed by inactivation at 95°C for 1 min.

A 2 µl aliquot of the cDNA products was used as template in a 50 µl PCR with 1× Q5 reaction buffer (NEB), 25 pmol of forward primers [18S_h34_fwd (5′-AAGGAATTGACGGAAGGGC-3′) or 18S_h45_fwd (5′-CGTCGCTAGTACCGATTGAATGGCTTAG-3′)] and 25 pmol of the respective reverse primers used for the reverse transcription (18S_h34_rev or 18S_h45_rev, respectively), 10 nmol dNTP mix, and 2.5 U of Q5 Polymerase (NEB). PCR was performed with an initial denaturation at 98°C for 30 s; followed by 34 cycles of denaturation at 98 °C for 10 s, annealing at 66 °C (helix 34) or 71°C (helix 45) for 30 s, elongation at 72 °C for 30 s; and a final elongation at 72°C for 2 min.

For Sanger sequencing, 5 µl of each PCR product was cleaned up using the Exo-CIP Rapid PCR Cleanup Kit (NEB) and sequenced using the forward PCR primer. Processed sequencing traces were viewed using Chromas.

## Results

### Sni445 is a novel ribosome assembly factor

**Figure 1.**
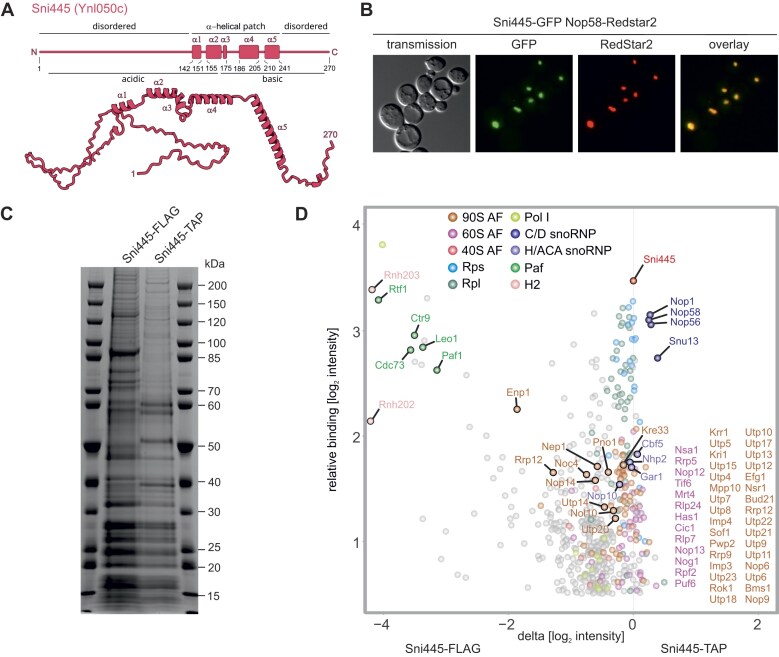
Sni445 is a novel ribosome assembly factor. (**A**) Secondary structure (upper panel) and AlphaFold [28] prediction (lower panel) of Sni445 (Ynl050c). (**B**) Fluorescence microscopy reveals that Sni445-GFP localizes to the nucleolus. Nop58–RedStar2 was used as a nucleolar marker. Overlay images show co-localization of Nop58-RedStar2 with Sni445-GFP. (**C**) Sni445-TAP and Sni445-FLAG eluates were analyzed by SDS-PAGE and Coomassie blue staining. (**D**) Sni445 co-purifies with ribosome assembly factors and box C/D snoRNA core proteins. Sni445-FLAG and Sni445-TAP eluates were analyzed by label-free MS in three biological replicates. *z*-score-normalized LFQ values were log_2_-transformed and visualized. The *x*-axis [(delta (log_2_ intensity)] shows the difference of averaged log_2_ intensity values of [Sni445-TAP]–[Sni445-FLAG] normalized to the Sni445 protein. The *y*-axis [relative binding (log_2_ intensity)] shows the averaged median-normalized label-free quantification from all experiments. Only specifically bound proteins with values >0.5 are shown (552 out of 2869). Proteins are annotated through color labels: Sni445 (red); box C/D proteins (C/D; 4, blue); box H/ACA proteins (H/ACA; 4, purple); polymerase-associated factor complex (Paf; 5, green); RNase H2 (H2, light pink); polymerase I subunits (Pol I; 11 light yellow); 90S assembly factors (90S AF; 65, brick); 60S assembly factors (60S AF; 40, magenta); 40S assembly factors (40S AF; 6, pink); small subunit r-proteins (RPS; 23, cyan); large subunit r-proteins (RPL; 45, turquoise).

A previously conducted TAP-based screen for the identification of proteins co-purifying with r-proteins also revealed, in addition to components of ribosomes and pre-ribosomal particles, several uncharacterized proteins, including Ynl050c, which we subsequently named Sni445 (see below) [27]. The observation that Sni445 was co-purified with TAP-tagged versions of multiple r-proteins, including Rps10 (eS10), Rps12 (eS12), Rps13 (uS15), Rps14 (uS11), Rps18 (uS13), Rps19 (eS19), Rps20 (uS10), Rps24 (eS24), and Rps31 (eS31) [27], indicates that Sni445 binds to complexes containing these r-proteins, either mature ribosomes or pre-ribosomal particles. Sni445 is predicted to be an unstructured protein with a negatively charged N- and a positively charged C-terminal part (Fig. 1A). The middle part of Sni445 (amino acids 142–241) harbors an α-helical patch with no defined domain structure, while the rest of the protein is highly disordered.

To distinguish between a function for Sni445 bound to mature ribosomes or as part of pre-ribosomal particles, we analyzed the subcellular localization of Sni445, based on the rationale that translation occurs in the cytoplasm, whereas ribosome biogenesis predominantly takes place in the nucleus. Fluorescence microcopy revealed that Sni445-GFP co-localizes with the nucleolar marker Nop58-RedStar2, indicating that its steady-state localization is in the nucleolus (Fig. 1B), suggesting a role for Sni445 in early steps of ribosome assembly. Several ribosome assembly factors show a nucleolar steady-state localization, but shuttle between the nucleus and cytoplasm [29, 30]. To assess whether Sni445-GFP is a shuttling protein or an exclusively nuclear protein, we employed the anchor-away technique [20]. Sni445 was fused to FRB-GFP in a rapamycin-resistant *tor1-1* strain, in which the plasma membrane-located protein Pma1 was fused to FKBP12 (human FK506-binding protein). Upon rapamycin treatment, a ternary complex between FRB, FKBP12, and rapamycin is formed, leading to the sequestration of FRB-GFP-tagged proteins at the plasma membrane if the protein enters the cytoplasm in the course of its functional cycle [20, 29]. Many known shuttling proteins relocalize to the plasma membrane within ∼15 min of rapamycin treatment [30]. In contrast, Sni445-FRB-GFP remained nucleolar even after 30 min of rapamycin treatment (Supplementary Fig. S2). A faint plasma membrane signal only became detectable after 60 min, probably due to *de novo* protein synthesis of Sni445-FRB-GFP. Supporting this, co-treatment with cycloheximide to block protein synthesis prevented plasma membrane localization, confirming that Sni445 does not shuttle and is an exclusively nucleolar protein (Supplementary Fig. S2).

To obtain first evidence for an association of Sni445 with early pre-ribosomal particles, we purified distinct pre-ribosomal intermediates and assessed the presence of Sni445 in comparison with purifications from control strains lacking *SNI445* due to chromosomal deletion. Indeed, Sni445 was among the proteins that were co-enriched with early 90S particles purified via Pwp2-TAP and, albeit only at low levels, with particles corresponding to later 90S and pre-40S maturation stages purified via Enp1-TAP (Supplementary Fig. S3A, B), suggesting that Sni445 is a previously unrecognized component of 90S pre-ribosomal particles.

To determine in more detail the stage of ribosome assembly in which Sni445 functions, we analyzed its protein interactome. Sni445 was fused to either a C-terminal TAP- or FLAG-tag and affinity purified from exponentially grown yeast cells. SDS-polyacrylamide gel electrophoresis (PAGE) followed by Coomassie blue staining revealed fewer protein bands in the TAP eluate than in the FLAG eluate (Fig. 1C). This could reflect reduced contamination due to the two-step TAP purification or, alternatively, a disruption of protein–protein interactions by the bulky TAP-tag or the longer purification procedure. MS showed that multiple components of pre-ribosomal particles co-purified with both Sni445-TAP and Sni445-FLAG, including r-proteins, 90S particle assembly factors (e.g. Krr1, Kri1, Utp5, and Utp22), and pre-60S particle factors, such as Nsa1 and Nop12. Most notably, and consistent with a recent high-throughput study [29], we found all four core proteins of box C/D snoRNPs, i.e. Nop1, Nop56, Nop58, and Snu13, strongly enriched in both the Sni445-TAP and Sni445-FLAG eluates, while box H/ACA snoRNP core proteins (Cbf5, Nhp2, Gar1, and Nop10) were also detected, but were not particularly enriched (Fig. 1D). In addition, several proteins were co-enriched with Sni445-FLAG compared with Sni445-TAP, suggesting that the large TAP tag might disturb certain interactions. In particular, Rtf1, Ctr9, Leo1, and Paf1, all components of the polymerase-associated factor (Paf) complex, which is involved in RNA polymerase II transcription, and the 3′ end formation of mRNAs and snoRNAs [31–33], as well as RNA polymerase I transcription [34, 35], co-purified with Sni445-FLAG (Fig. 1D). Additionally, RNase H2 subunits Rnh202 (H2B subunit) and Rnh203 (H2C subunit) were also strongly co-purified (Fig. 1D).

Based on the co-purification of multiple ribosome assembly factors with Sni445-TAP and Sni445-FLAG, together with the reciprocal co-purification of Sni445 with Pwp2-TAP and Enp1-TAP particles, we conclude that Sni445 functions as a ribosome assembly factor. The pronounced co-enrichment of box C/D snoRNP proteins with Sni445 further suggests that Sni445 may act in the context of box C/D snoRNP-mediated steps during ribosome biogenesis.

### Sni445 is a component of the snR4 and snR45 acetylation guide snoRNPs

**Figure 2.**
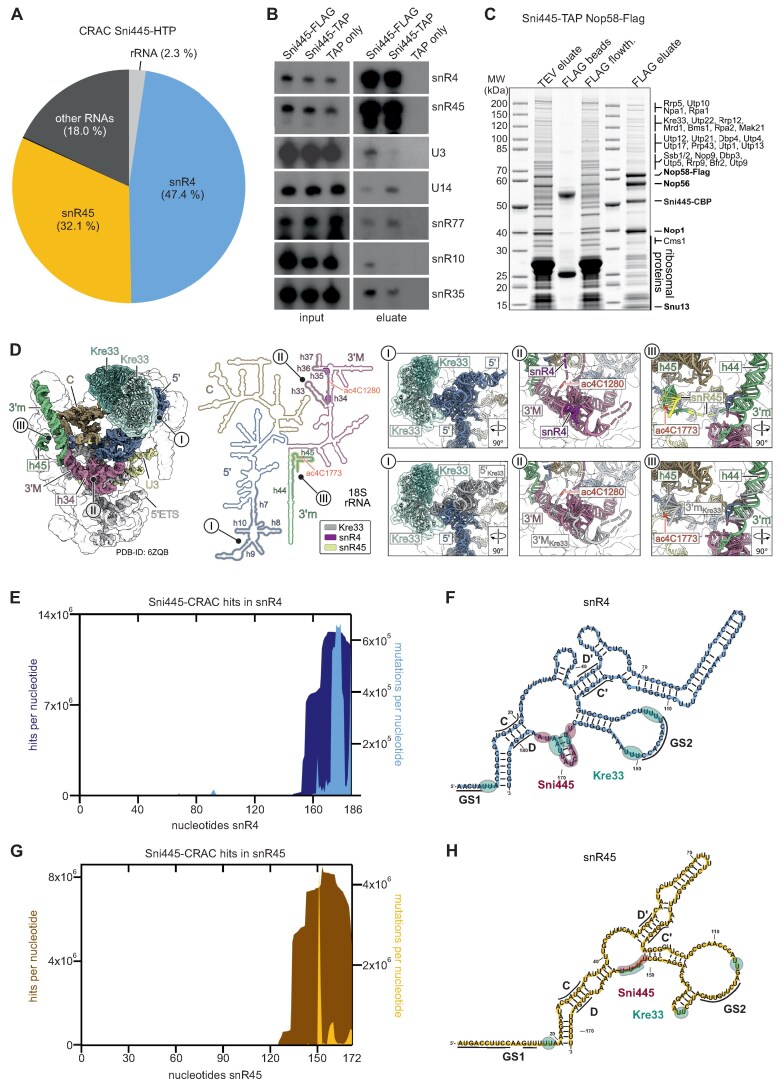
Sni445 associates with the acetylation guide snoRNAs snR4 and snR45. (**A**) CRAC analysis reveals that Sni445 associates with the box C/D snoRNAs snR4 (blue) and snR45 (dark yellow). The pie chart shows RNA species cross-linked with Sni445-HTP. See also Supplementary Fig. S4A for comparison with the negative control. (**B**) snR4 and snR45 are strongly co-enriched with Sni445. RNA was extracted from Sni445-TAP, Sni445-FLAG, or TAP-only eluates. Northern blot analysis was performed on inputs and eluates using probes specific for snR4 and snR45, other box C/D snoRNAs U3, U14, and snR77, and box H/ACA snoRNAs snR10 and snR35. The inputs and eluates were analyzed on the same gel, and the same exposures are shown, allowing direct comparisons. (**C**) Split-tag purification of complexes containing both Sni445 and Nop58. Complexes were first purified via Sni445-TAP followed by Nop58-FLAG, and analyzed by SDS-PAGE and Coomassie blue staining. The TEV eluate (all proteins co-purified with Sni445-TAP), the FLAG flowthrough (unbound fraction), and the FLAG bead fraction (proteins not eluted by FLAG peptide) were loaded as controls. Proteins identified by MS are indicated. (**D**) Structure of a 90S pre-ribosome in state B2 (PDB 6ZQB, [37]), shown alongside the secondary structure of the mature 18S rRNA. The Kre33 dimer is highlighted in turquoise, and the structural domains of the 18S rRNA are color coded: 5′ domain in blue; central domain in brown; 3′ major domain in magenta; and 3′ minor domain in green. The U3 snoRNA is shown in bright yellow. Kre33 cross-linking sites identified by CRAC (II) are indicated in the 3D and 2D structure (I for the cross-linking site in the 5′ domain, II for the site in helix h34, and III for the site in helix h45). Detailed views of these regions are provided in the zoom-in panels. See also Supplementary Fig. S1B for a more detailed depiction of snR4 and snR45 base-pairing with rRNA, and Supplementary Fig. S5 for a more detailed representation of the 18S rRNA secondary structure. (**E**) Distribution of sequence reads (dark blue) and mutations/deletions (light blue) in snR4 recovered in the Sni445-HTP CRAC experiment. The left *y*-axis shows read counts; the right *y*-axis shows mutation frequencies. Mutations arise from cross-link-induced reverse transcription errors and thus mark cross-linking sites. (**F**) Secondary structure of snR4 (blue) (II). snR4 contains two guide sequences (GS1 and GS2, black lines) that base-pair with 18S rRNA, and conserved box C/D and box C′/D′ motifs (black lines). Sni445 cross-linking sites, identified by mutations/deletions in the sequencing reads, are marked by red bubbles; major Kre33 cross-linking hits (II) are indicated by green bubbles. (**G**) Distribution of sequence reads (brown; left *y*-axis) and mutations (dark yellow; right *y*-axis) in snR45 from the Sni445-HTP CRAC experiment. (**H**) Predicted secondary structure of snR45 (yellow). snR45 harbors two guide sequences (GS1 and GS2, black lines) that base-pair with 18S rRNA and conserved box C/D and box C′/D′ motifs (black lines). Sni445 cross-linking sites, identified by mutations/deletions in the sequencing reads, are marked by red bubbles; major Kre33 cross-linking hits (II) are indicated by turquoise bubbles.

Based on its co-purification with pre-ribosomal particles and box C/D snoRNP proteins, we speculated that Sni445 might interact with pre-rRNA and/or snoRNAs. To identify RNA sequences bound by Sni445, we performed RNA CRAC [36]. A strain expressing Sni445 fused to an HTP tag, as well as a wild-type control strain, were subjected to *in vivo* UV-cross-linking, and Sni445 and its associated RNAs were purified and partially digested with RNases. RNA fragments bound to Sni445 were isolated, reverse transcribed, PCR-amplified, and the resulting cDNA library was subjected to deep sequencing. The obtained sequences were mapped to the yeast genome and the proportions of reads derived from the different RNA families were analyzed. Strikingly, ∼80% of the sequence reads mapped to snoRNAs, with the vast majority corresponding to only two snoRNAs, the box C/D snoRNAs snR4 (∼47% of all reads) and snR45 (∼32% of all reads) (Fig. 2A; Supplementary Fig. S4A). These two snoRNAs do not guide methylation, but instead guide *N*^4^-acetylation of cytidines C1280 and C1773 in the 18S rRNA via the acetyltransferase Kre33. These findings prompted us to name the protein Sni445 (snoRNA interactor of snR4 and snR45). To confirm this result, we performed northern blotting analyses on eluates from Sni445-TAP and Sni445-FLAG purifications. We observed a strong enrichment of snR4 and snR45 compared with other box C/D and box H/ACA snoRNAs, further confirming their specific association with Sni445 (Fig. 2B, compare signals in inputs and eluates).

To further characterize the complex(es) containing Sni445 and the snR4/snR45 snoRNPs, we employed a split two-step affinity purification strategy. First, Sni445-TAP was purified, followed by a second purification step using the box C/D core protein Nop58 tagged with a FLAG-tag as bait. This strategy yielded stoichiometric amounts of Sni445 along with the canonical box C/D snoRNP core proteins Nop1, Nop56, and Nop58 (Fig. 2C), indicating that Sni445 forms a stable complex with box C/D snoRNPs. In addition, several substoichiometric bands corresponding to 90S assembly factors, including Rrp5, Utp10, Utp22, Rrp12, and Kre33, as well as pre-60S assembly factors such as Npa1 and Mak21, were detected (Fig. 2C), suggesting that a subset of the Sni445-containing box C/D complexes are associated with early pre-ribosomal particles. Taken together, these data demonstrate that Sni445 is a bona fide component of the snR4 and snR45 snoRNPs, probably associating with them already prior to their engagement with pre-ribosomal particles. The presence of 90S assembly factors in the purifications further suggests that Sni445 remains associated with these snoRNPs when they interact with 90S pre-ribosomal particles.

The snoRNAs snR4 and snR45 guide site-specific acetylation by base-pairing with distinct regions of the 18S rRNA (Fig. 2D; Supplementary Figs S1B and S5). Specifically, snR4 targets helix h34 in the 3′ major domain, to guide acetylation at C1280 (II in Fig. 2D), and snR45 pairs with helix h45 in the 3′ minor domain, near the 3′ end of the 18S rRNA, guiding acetylation at C1773 (III in Fig. 2D). Models based on secondary structure predictions and dimethyl sulfate (DMS) footprinting data suggest that both snoRNAs contain two guide sequences (GS1 and GS2), interacting with 18S rRNA sequences upstream and downstream of the cytidines to be modified (Fig. 2F, H; Supplementary Fig. S1B, II). Interestingly, structural analyses of 90S particles show that Kre33, the enzyme responsible for catalyzing both modifications [11, 12], binds to the 5′ domain of the 18S rRNA (Fig. 2D, I), a region distant from helices h34 (II) and h45 (III) [37]. However, Kre33 CRAC data revealed not only the 18S rRNA-binding site visible in the 90S structures, but also additional binding sites in the vicinity of the cytidines in 18S rRNA helices h34 and h45 targeted for acetylation, as well as binding sites in snR4 and snR45 (Fig. 2D, F, H; Supplementary Fig. S5, II). These extra binding sites compared with the cryo-electron microscopy (EM) structures suggest that conformational re-arrangements take place within 90S particles during the assembly process to position Kre33 at its target nucleotides.

Mapping of Sni445 CRAC reads onto the snR4 and snR45 sequences revealed that Sni445 binds both snoRNAs at their 3′ regions, near the conserved D boxes (Fig. 2E, 2G, sequencing reads marked in dark blue and dark yellow). Many reads contained deletions or substitutions, indicating that these residues correspond to the actual cross-linking sites (highlighted in light blue in Fig. 2E and yellow in Fig. 2G, and marked as Sni445-binding sites in Fig. 2F and H). Notably, these binding sites are in very close proximity to those previously identified in Kre33 CRAC experiments (Fig. 2F, H). In addition, a fraction of CRAC reads extended ∼10–11 nucleotides beyond the 3′ ends of snR4 and snR45 (Supplementary Fig. S4B, C), suggesting that a subpopulation of Sni445 associates with immature snR4 and snR45 that have not yet undergone their final processing to the mature 3′ end. This indicates that Sni445 engages with snR4 and snR45 probably co-transcriptionally and remains a stable component of the snoRNPs that are then incorporated into 90S pre-ribosomes.

### SNI445 deletion phenocopies the effects of snR4 and snR45 deletions

**Figure 3.**
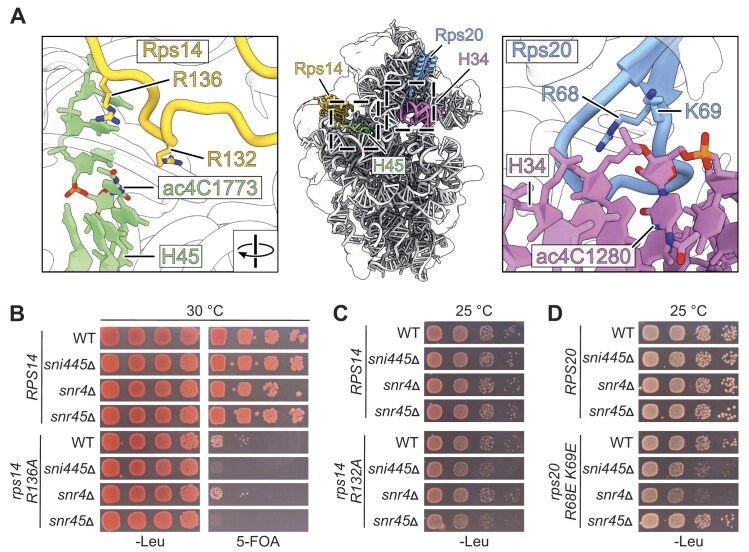
Deletion of *SNI445* phenocopies deletion of *SNR4* or *SNR45*. (**A**) Structure of the small ribosomal subunit (PDB 8CGN) [38]. 18S rRNA (gray), Rps14 (light orange), and Rps20 (blue) are labeled and displayed as cartoon representations. 18S rRNA helices h34 and h45 are highlighted. The zoom-ins highlight the positions of ac4C1280, located near residues R68 and K69 of Rps20, and ac4C1773, located near residues R132 and R136 of Rps14. (**B**) Synthetic lethality between *rps14a.R136A* and deletion of *SNI445* or *SNR45*. Yeast cells (*rps14a*∆ *rps14b*∆) carrying a *URA3* plasmid with wild-type (WT) *RPS14A* and *LEU2* plasmids with either WT *RPS14A* or the mutant *rps14a.R136A* allele, combined with *SNI445, SNR4*, or *SNR45* deletion, were spotted in serial dilutions on SDC-Leu and 5-FOA-containing plates and incubated for 3 days at 30°C. Lack of growth on 5-FOA plates indicates failure to lose the *URA3*-*RPS14A* plasmid, suggesting synthetic lethality. (**C**) Genetic enhancement of the mild growth defect of the *rps14.R132A* mutant upon deletion of *SNI445* or *SNR45*. Yeast cells (*rps14a*∆ *rps14b*∆) carrying *LEU2* plasmids with either the WT *RPS14* or mutant *rps14a.R132A* allele, combined with *SNI445, SNR4*, or *SNR45* knockout, were spotted in serial dilutions on SDC-Leu plates and incubated at 25°C for 2 days. These results are compared with results from additional incubation temperatures (30°C and 37°C) in Supplementary Fig. S6B. (**D**) Genetic enhancement of the growth defect of the *rps20.R68E/K69E* mutant upon deletion of SNI445 or SNR4. Yeast cells (*rps20*∆) carrying *LEU2* plasmids with either WT RPS20 or the mutant *rps20.R68E/K69E* allele, either in an *SNI445* WT background or combined with a *SNI445, SNR4*, or *SNR45* knockout, were spotted in serial dilutions on SDC-Leu plates and incubated at 25°C for 3 days. These results are compared with results from additional incubation temperatures (30°C and 37°C) in Supplementary Fig. S6C.

*SNI445* is not essential, and its absence does not cause any significant rRNA processing defects (Supplementary Fig. S6A). We speculated, however, that combined mutation of *SNI445* and genes encoding proteins that function together with or in proximity to Sni445 would result in synergistic growth defects. Inspection of the 3D structure of the 40S subunit [38] revealed that the rRNA nucleotides that are acetylated by Kre33 lie in close physical proximity to r-proteins. ac4C1773 in 18S rRNA helix h45 is in proximity to the C-terminal tail of Rps14 (Fig. 3A, [38]), and ac4C1280 in 18S rRNA helix h34 is positioned in close proximity to charged residues in a flexible loop of Rps20 (Fig. 3A). Notably, mutants of both the flexible loop of Rps20 and the C-terminal tail of Rps14 were shown to display defects in late stages of 40S ribosomal subunit biogenesis [39, 40].

To test for genetic interactions between *RPS14* and *SNI445, SNR4*, or *SNR45*, we used strains lacking the chromosomal copies of *RPS14A* and *RPS14B*, complemented with a *URA3* plasmid carrying wild-type *RPS14A*, and additionally carrying deletions of *SNI445, SNR4*, or *SNR45*. These strains were transformed with *LEU2* plasmids harboring either the *rps14a.R136A* allele, leading to a charge neutralizing substitution in the C-terminal tail, or the wild-type *RPS14A* as a control. The effects of the *LEU2* plasmid-borne alleles were assessed by growth on 5-FOA plates, which select for cells that have lost the complementing *URA3* plasmid (Fig. 3B). Cells containing the *rps14a.R136A* allele as the sole source of *RPS14* (visible on 5-FOA plates) formed small colonies, indicating a strong growth defect. Notably, combining *rps14a.R136A* with either *snr45*Δ or *sni445*Δ resulted in a complete failure of cells to grow on 5-FOA, suggesting synthetic lethality, while no such genetic interaction was observed when the *rps14a.R136A* mutations was combined with *snr4*Δ (Fig. 3B). To validate these genetic interactions with a milder allele, we generated *rps14a.R132A*, which did not show a growth defect on its own (Fig. 3C). Both the *rps14.R132A sni445*Δ and the *rps14.R132A snr45*Δ double mutant displayed smaller colonies at 25°C than the corresponding single mutants, indicating a synthetic growth defect. In contrast, *SNR4* deletion did not affect growth of the *rps14.R132A* mutant (Fig. 3C; Supplementary Fig. S6B).

In analogy, combining the *rps20.R68E/K69E* allele, leading to charge reversal exchanges in the Rps20 flexile loop in proximity to the snR4-binding site, with either *sni445*Δ or *snr4*Δ, resulted in a synthetic growth defect at 25°C, suggesting that *RPS20* genetically interacts with both *SNI445* and *SNR4*. In contrast, no genetic interaction was observed between *RPS20* and *SNR45* (Fig. 3D; Supplementary Fig. S6C).

In summary, *SNI445* deletion phenocopies the effects of *SNR4* and *SNR45* deletion with respect to the genetic interactions with *RPS20* and *RPS14*, respectively. These results strongly suggest that the genetic interactions observed for *SNI445* arise from reduced recruitment and/or function of the acetylation guide snoRNAs snR4 and snR45.

### Sni445 physically interacts with acetyltransferase Kre33

**Figure 4.**
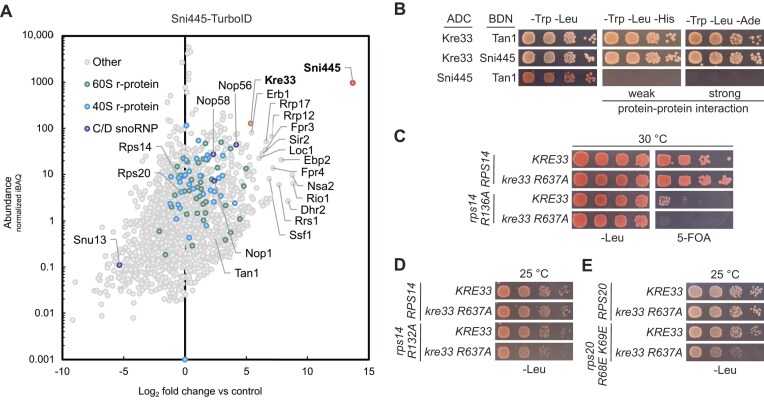
Sni445 interacts with the acetyltransferase Kre33. (**A**) Physical proximities of Sni445 revealed by TurboID-based proximity labeling. For each protein detected in the streptavidin pull-down of cells expressing Sni445-TurboID, the normalized abundance (iBAQ, intensity-based absolute quantification; *y*-axis) is plotted against its relative enrichment (log_2_-transformed; *x*-axis) compared with two negative control purifications (GFP-TurboID and NLS-GFP-TurboID baits, accounting for cytoplasmic and nuclear backgrounds, respectively). Proteins enriched relative to the controls can be found on the right side of the plot. (**B**) Y2H interaction assays between Sni445, Kre33, and the tRNA acetylation adaptor Tan1. Proteins were fused C-terminally to the Gal4 activation domain (ADC) or N-terminally to the Gal4 DNA-binding domain (BDN). Cells were spotted in serial dilutions on SDC-Trp-, -Leu plates (growth control), SDC-Trp, -Leu, -His plates (growth indicates weak interaction), and SDC-Trp, -Leu, -Ade plates (growth indicates strong interaction), and incubated for 3 days at 30°C. Combinations with empty vectors expressing the non-fused ADC or BDN were tested as negative controls and are shown in Supplementary Fig. S7. (**C**) Catalytic inactivation of Kre33 (*kre33.R637A*) results in synthetic lethality with the *rps14a.R136A* mutation. Yeast cells (*rps14a*∆ *rps14b*∆) carrying a *URA3* plasmid with wild-type *RPS14A*, and *LEU2* plasmids with either wild-type *RPS14A* or *rps14a.R136A* in combination with wild-type *KRE33* or the genomically integrated *kre33.R637A* allele were spotted in a serial dilution on SDC-Leu and 5-FOA-containing plates, and incubated for 3 days at 30°C. No growth on 5-FOA-containing plates indicates failure to lose the *URA3*-*RPS14A* plasmid, suggesting synthetic lethality. (**D**) The *kre33.R637A* mutation enhances the mild growth defect of the *rps14a.R132A* mutant. Yeast cells (*rps14a*∆ *rps14b*∆) carrying *LEU2* plasmids with either wild-type *RPS14A* or the *rps14a.R132A* allele in combination with wild-type *KRE33* or the genomically integrated *kre33.R637A* allele were spotted in serial dilutions on SDC-Leu plates and incubated for 2 days at 25°C. These results are compared with results from additional incubation temperatures (30°C and 37°C) in Supplementary Fig. S6D. (**E**) The catalytically inactive *kre33.R637A* mutant enhances the growth defect of the *rps20.R68E/K69E* mutant. Yeast cells (*rps20*∆) carrying *LEU2* plasmids with either wild-type *RPS20* or the *rps20.R68E/K69E* allele in combination with wild-type *KRE33* or the genomically integrated *kre33.R637A* allele, encoding a catalytic inactive variant, were spotted in serial dilutions on SDC-Leu plates and incubated for 3 days at 25°C. These results are compared with results from additional incubation temperatures (30°C and 37°C) in Supplementary Fig. S6E.

We hypothesized that Sni445 associates with 90S particles primarily through snR4 and snR45, and potentially in addition via protein–protein interactions with ribosome assembly factors. To identify potential protein docking sites of Sni445, we performed TurboID-based proximity labeling [41, 42]. This method is based on the fusion of a bait protein to TurboID, an improved variant of the promiscuous biotin ligase BirA*, which efficiently biotinylates proteins that are in close physical proximity to the enzyme. Biotinylated proteins are then affinity-purified via streptavidin beads and identified by MS. As expected, box C/D core proteins Nop56, Nop58, and Nop1 were enriched, with Nop56 showing the highest abundance and strongest enrichment. Among the prominently enriched ribosome assembly factors, there were factors associated with or acting on 90S/pre-40S particles, such as Kre33, Rrp12, Dhr2, and Rio1, but also pre-60S factors, including Erb1, Loc1, Nsa2, Rrp17, and Ssf1 (Fig. 4A).

The strong enrichment of Kre33, the acetyltransferase responsible for catalyzing the modifications guided by snR4 and snR45, together with the observation that both Sni445 and Kre33 [11] interact with the snR4 and snR45 snoRNAs in close proximity (Fig. 2F, H), suggested that Sni445 might directly interact with Kre33. Interestingly, Kre33 also modifies tRNAs, a process assisted by the adaptor protein Tan1 [15]. However, neither Tan1 nor other tRNA-binding proteins was particularly enriched in the Sni445 TurboID experiment (Fig. 4A), suggesting that Sni445 interacts with Kre33 specifically in the context of pre-ribosomal particles. To obtain further evidence for a physical interaction between Sni445 and Kre33, we performed Y2H assays, in which growth on plates lacking histidine or adenine indicates weak or strong interactions, respectively. Importantly, Y2H assays revealed that Sni445 strongly interacts with Kre33, to a similar extent to its interaction with Tan1. In contrast, and as expected, Sni445 did not interact with Tan1 (Fig. 4B; Supplementary Fig. S7).

We next wondered if the phenotypes of *SNI445, SNR4*, and *SNR45* deletion, apparent from their genetic interactions with *rps20* and *rps14* alleles, are the consequence of the missing acetyl groups at C1280 and C1773 or, alternatively, are due to a modification-independent function of snR4 and snR45. To distinguish between these two possibilities, we employed a catalytically inactive *kre33* mutant, in which the Kre33 protein carries an amino acid exchange at position 637 (R > A) [15]. Notably, the *kre33.R637A* mutant displayed synthetic lethality when combined with the *rps14a.R136A* allele (Fig. 4C) and a synthetic growth defect when combined with the *rps14a.R132A* allele (Fig. 4D; Supplementary Fig. S6D), thus recapitulating the effects of *SNI445* or *SNR45* deletion (Fig. 3B, C; Supplementary Fig. S6B).

Similarly, a synthetic growth defect could be observed when the *kre33.R637A* allele was combined with the *rps20.R68E/K69E* allele (Fig. 4E; Supplementary Fig. S6E), phenocopying the impact of *SNI445* or *SNR45* deletions (Fig. 3D; Supplementary Fig. S6C). The observation that loss of the catalytic activity of Kre33 is sufficient to explain the synthetic growth defects is strong evidence that all observed genetic interactions result from loss of specific rRNA acetylation events.

### Sni445 recruits snR4 and snR45 to 90S particles to guide acetylation by Kre33

**Figure 5.**
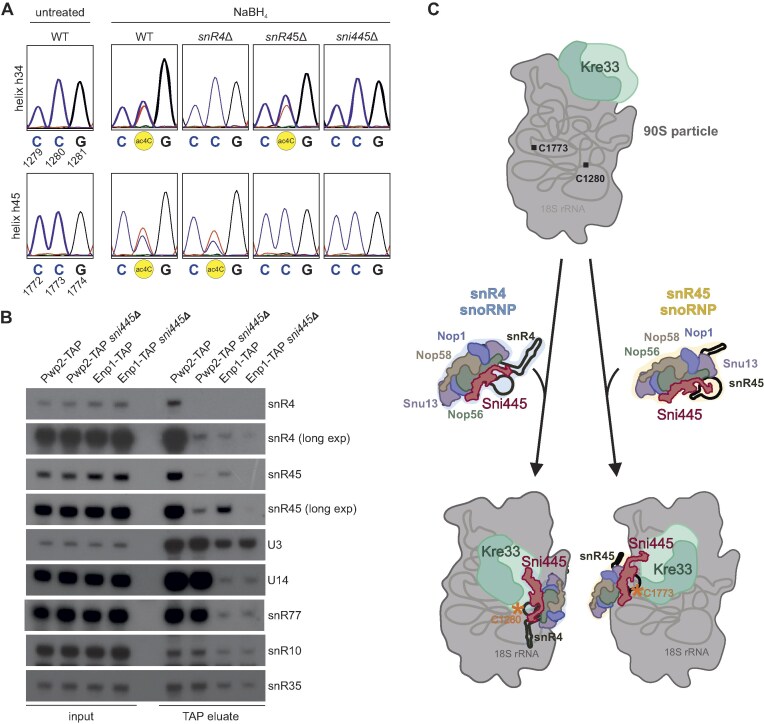
Sni445 recruits snR4 and snR45 to pre-ribosomal particles to facilitate acetylation by Kre33. (**A**) Sni445 is essential for acetylation at C1280 and C1773. Sanger sequencing analysis of PCR-amplified cDNAs generated from ac4C RNAs following NaBH_4_ treatment and reverse transcription. The red trace in the chromatograms represents Ts. Acetylated Cs are apparent from combined C/T peaks in NaBH_4_-treated samples. (**B**) Deletion of *SNI445* alters the snoRNA composition of pre-ribosomal particles. RNA was extracted from TAP eluates of early 90S (Pwp2-TAP) and late 90S/pre-40S (Enp1-TAP) particles purified from wild-type and *sni445*Δ strains. Northern blot analysis was performed on inputs and TAP eluates using probes specific for snR4, snR45, U3, snR35, and snR10. Asterisks indicate residual signals from previously hybridized probes that were not fully removed during stripping. (**C**) Model of the function of Sni445 in recruiting snR4 and snR45 to 90S particles to guide acetylation by Kre33. Free snR4 and snR45 snoRNPs containing Sni445 associate with 90S particles that already contain Kre33. A subsequent structural rearrangement, potentially involving repositioning of Kre33 as depicted, enables the interaction of Kre33 with Sni445 and the snoRNAs snR4 and snR45 to place Kre33 in the appropriate orientation for substrate acetylation.

As Sni445 is a stable component of the snR4 and snR45 snoRNPs and interacts with the acetyltransferase Kre33, we next asked whether it is required for efficient acetylation at C1280 and C1773. To test this, we employed a previously established reverse transcription-based assay to detect ac4C [26]. This method relies on the reduction of ac4C by sodium borohydride (NaBH_4_), resulting in conversion to *N*^4^-acetyl-3,4,5,6-tetrahydrocytidine. This modification causes reverse transcriptases to misincorporate nucleotides, most notably adenosine instead of guanosine, producing mixed C/T peaks in the sequencing readout following cDNA amplification. Consistent with previous findings [26, 43], partial misincorporation was observed at both C1280 and C1773 in NaBH_4_-treated, but not in untreated wild-type samples (Fig. 5A). In contrast, misincorporation was absent at C1280 in the *snr4*Δ strain and at C1773 in the *snr45*Δ strain, confirming loss of acetyl modifications at these sites. Strikingly, both modification signals were fully abolished in the *sni445*Δ strain, indicating that Sni445 is essential for snR4- and snR45-guided cytidine acetylation (Fig. 5A).

Importantly, abolished acetylation in the absence of Sni445 is not caused by a reduced association of Kre33 with 90S particles, as Kre33 was co-purified equally well with the Pwp2-TAP and Enp1-TAP baits irrespective of the presence or absence of Sni445 (Supplementary Fig. S3A, B), indicating that the abolished C1280 and C1773 acetylation in the *sni445*Δ strain is not a consequence of failed Kre33 recruitment. Another possibility could be that Sni445 is important for snR4 and snR45 association with pre-ribosomal particles. To test this possibility, we affinity-purified early 90S particles using Pwp2-TAP and late 90S/pre-40S particles using Enp1-TAP as baits and assessed the effects of *SNI445* deletion on the association of snR4 and snR45 by northern blotting (Fig. 5B). Importantly, the steady-state levels of snR4 and snR45 were unaffected by *SNI445* deletion, as shown by the input controls. In wild-type cells, both snoRNAs were detected in Pwp2-purified particles and, to a lesser extent, in Enp1-purified particles (Fig. 5B). However, in the *sni445*Δ strains, the association of snR4 and snR45 with these pre-ribosomal particles was substantially reduced, whereas the binding of other snoRNAs (i.e. box C/D snoRNAs U3, U14, and snR77 and box H/ACA snoRNAs snR35 and snR10) remained unchanged (Fig. 5B). Together, our findings suggest that Sni445 promotes the recruitment or stabilization of snR4 and snR45 within 90S particles, thereby enabling Kre33 to catalyze acetylation at C1280 and C1773 (Fig. 5C).

## Discussion

In this study, we identify Sni445 as a novel ribosome assembly factor and a previously unrecognized auxiliary component of the snR4 and snR45 box C/D snoRNPs. Unlike canonical box C/D snoRNPs, which guide 2′-*O*-methylation, snR4 and snR45 guide *N*^4^-acetylation of 18S rRNA residues C1280 and C1773, respectively, via the acetyltransferase Kre33 [11, 12].

Moreover, Sni445 appears to be also associated with members of the Paf1 complex (Fig. 1D), which functions in RNA polymerase II transcription elongation and in the recruitment of 3′-end-processing factors for snoRNAs [31, 32]. Consistent with this, we observed that a fraction of Sni445 cross-linked to 3′-extended forms of snR4 and snR45, indicating that Sni445 binds to these snoRNAs already during their synthesis. However, we did not detect extended snR4 or snR45 species in *sni445*Δ strains by northern blot analysis (Fig. 5B), suggesting that Sni445 does not contribute to 3′ end formation. Instead, its association with the Paf1 complex may reflect a role for this complex in recruiting Sni445 to nascent snoRNA transcripts.

We show that Sni445 is stably associated with free snR4 and snR45 snoRNPs (Fig. 2C), and remains associated with these upon their incorporation into pre-ribosomal particles, as demonstrated by the co-purification of Sni445 with ribosome assembly factors (Supplementary Fig. S3) and the reciprocal co-purification of ribosome assembly factors with Sni445 (Figs 1D and 4A).

Interestingly, although Kre33 also cross-links to both snoRNAs [11], and directly interacts with Sni445 (Fig. 4B), it is not present in free Sni445-containing snoRNP complexes (Fig. 2C), suggesting that Kre33 only interacts with these snoRNPs within 90S particles. Notably, Kre33 is an essential structural component of the 90S pre-ribosome [44–46], whereas snR4, snR45, and Sni445 are non-essential. This further suggests that Kre33 must associate with 90S particles independently of the snR4 and snR45 snoRNPs. Consistently, deletion of *SNI445* results in the loss of snR4 and snR45 from pre-ribosomal particles (Fig. 5B), while Kre33 levels remain unaffected (Supplementary Fig. S3).

Intriguingly, in available cryo-EM structures of 90S particles, Kre33 is positioned far from its acetylation target nucleotides C1280 and C1773 (Fig. 2D, [37, 44, 46, 47]). In contrast, previous CRAC analyses revealed that Kre33 binds not only to the region observed in the cryo-EM structures, helices h7–h10 of the 18S rRNA 5′ domain, but also to the regions where snR4 and snR45 base-pair with the rRNA, namely h33–h36 in the 3′ major domain and h44–h45 in the 3′ minor domain, respectively (Fig. 2D; Supplementary Fig. S5, II). These findings imply that the 90S intermediates captured by cryo-EM must undergo substantial rRNA rearrangements or Kre33 repositioning to allow Kre33 to engage with snR4 and snR45 and to bring the acetylation target nucleotides into the vicinity of its catalytic center. Notably, Sni445 and Kre33 physically interact with each other, and both proteins also interact with snR4 and snR45. This strongly suggests that Sni445 and Kre33 can bind the same snoRNP simultaneously. Mapping of the nucleotides cross-linked to Sni445 and Kre33 (Fig. 2F, H) suggests that Kre33 establishes multiple contacts with the snoRNAs, one of which is located very close to or partially overlaps with the Sni445 cross-linking site. It will be an interesting future endeavor to structurally characterize and compare the conformations of the snoRNPs when bound to only Sni445 or both Sni445 and Kre33, as well as determining whether Sni445 contributes to positioning snoRNA-bound target nucleotides to enable efficient modification by Kre33.

Strikingly, deletion of Sni445 abolishes acetylation at C1280 and C1773 (Fig. 5A) and dramatically reduces the association of snR4 and snR45 with 90S particles (Fig. 5B). This strongly suggests that Sni445 is required for the recruitment and/or stable anchoring of these snoRNPs to 90S particles. Hence, in the case of snR4 and snR45, the snoRNA guide sequences and canonical core proteins are not sufficient to mediate targeting of these snoRNAs, but additional recruitment mechanisms, involving Sni445, are required.

Beyond the Sni445-containing 90S complexes characterized here, our TAP and FLAG purification experiments also revealed co-enrichment of pre-60S assembly factors with Sni445 (Fig. 2C), a finding further supported by TurboID-based proximity labeling, which indicates physical proximity to pre-60S components (Fig. 4A). In addition, snR4 was previously shown to bind 5S rRNA (nt 22–32) and 25S rRNA (nt 1866–1878) [48]. Together, these observations raise the possibility that Sni445, potentially as part of snR4/snR45 snoRNPs, might also contribute to pre-60S biogenesis.

Interestingly, while canonical snoRNPs only contain four different core proteins, there are a few examples of atypical snoRNPs containing distinct additional protein components. For instance, Rrp9 is a component of the box C/D U3 snoRNP, Utp23, Kri1, and Krr1 of the box H/ACA snR30 snoRNP, while the Npa1 complex is associated with the box C/D snR190 snoRNP [13, 14, 49–51]. We now add Sni445 to this list of dedicated components of snoRNPs. Of note, all these atypical snoRNPs are involved in functions other than the canonical roles of their snoRNA classes in guiding methylation or pseudouridylation.

## Supplementary Material

gkag030_Supplemental_Files

## Data Availability

The MS proteomics raw data have been deposited to the ProteomeXchange Consortium via the PRIDE partner repository (https://www.ebi.ac.uk/pride/) with the dataset identifier PXD065447 for the label-free mass spectrometry data and PXD067909 for the TurboID-based proximity labeling data. Additionally, a file containing the MaxQuant output data as well as the processed data are provided as Supplementary Table S3. NGS analysis files of raw and processed data were deposited in the Gene Expression Omnibus database under the accession number GSE299803.

## Notes

Present address: Biotech Research and Innovation Center, Faculty of Health and Medical Sciences, University of Copenhagen, 2200 Copenhagen, Denmark
