# Supplementary material for: Sni445 recruits box C/D snoRNPs snR4 and snR45 to guide ribosomal RNA acetylation by Kre33
Source: Nucleic Acids Res. 2026 Jan 28;54(3):gkag030. doi: 10.1093/nar/gkag030 (PMC12848939; doi:10.1093/nar/gkag030)
Supplement: gkag030_Supplemental_Files [file gkag030_supplemental_files.zip › Hafneretal_SupplementaryData.pdf]

Supplementary Data

Sni445 recruits box C/D snoRNPs snR4 and snR45 to guide ribosomal RNA acetylation by Kre33

Jutta Hafner[1,7,8], Ingrid Zierler[1,7], Hussein Hamze[2], Sébastien Favre[3], Matthias Thoms[4], Natalia Kunowska[5], Sarah Rimser[1,7], Benjamin Albert[2], Tomas Caetano[6], Marion Aguirrebengoa[6], Roland Beckmann[4], Ulrich Stelzl[5,7], Dieter Kressler[3], Anthony K. Henras[2], Brigitte Pertschy[1,7+]

[+]Correspondence should be addressed to B.P. (brigitte.pertschy@uni-graz.at)

[1] Institute of Molecular Biosciences, University of Graz, 8010 Graz, Austria.

[2] Molecular Cellular and Developmental Biology Unit (MCD), Centre de Biologie Intégrative (CBI), Université de Toulouse, CNRS, UPS, 31062, Toulouse, France.

[3] Unit of Biochemistry, Department of Biology, University of Fribourg, 1700 Fribourg, Switzerland.

[4] Gene Center, University of Munich, 81377 Munich, Germany.

[5] Institute of Pharmaceutical Sciences, Pharmaceutical Chemistry, University of Graz, 8010 Graz, Austria.

[6] BigA Core Facility, Centre de Biologie Intégrative (CBI), Université de Toulouse, 31062 Toulouse, France.

[7] BioTechMed-Graz, Graz, Austria.

[8] Present address: Biotech Research and Innovation Center, Faculty of Health and Medical Sciences, University of Copenhagen, 2200 Copenhagen, Denmark.

Keywords: box C/D snoRNP, snR4, snR45, Kre33, cytidine acetylation, ribosome biogenesis, yeast

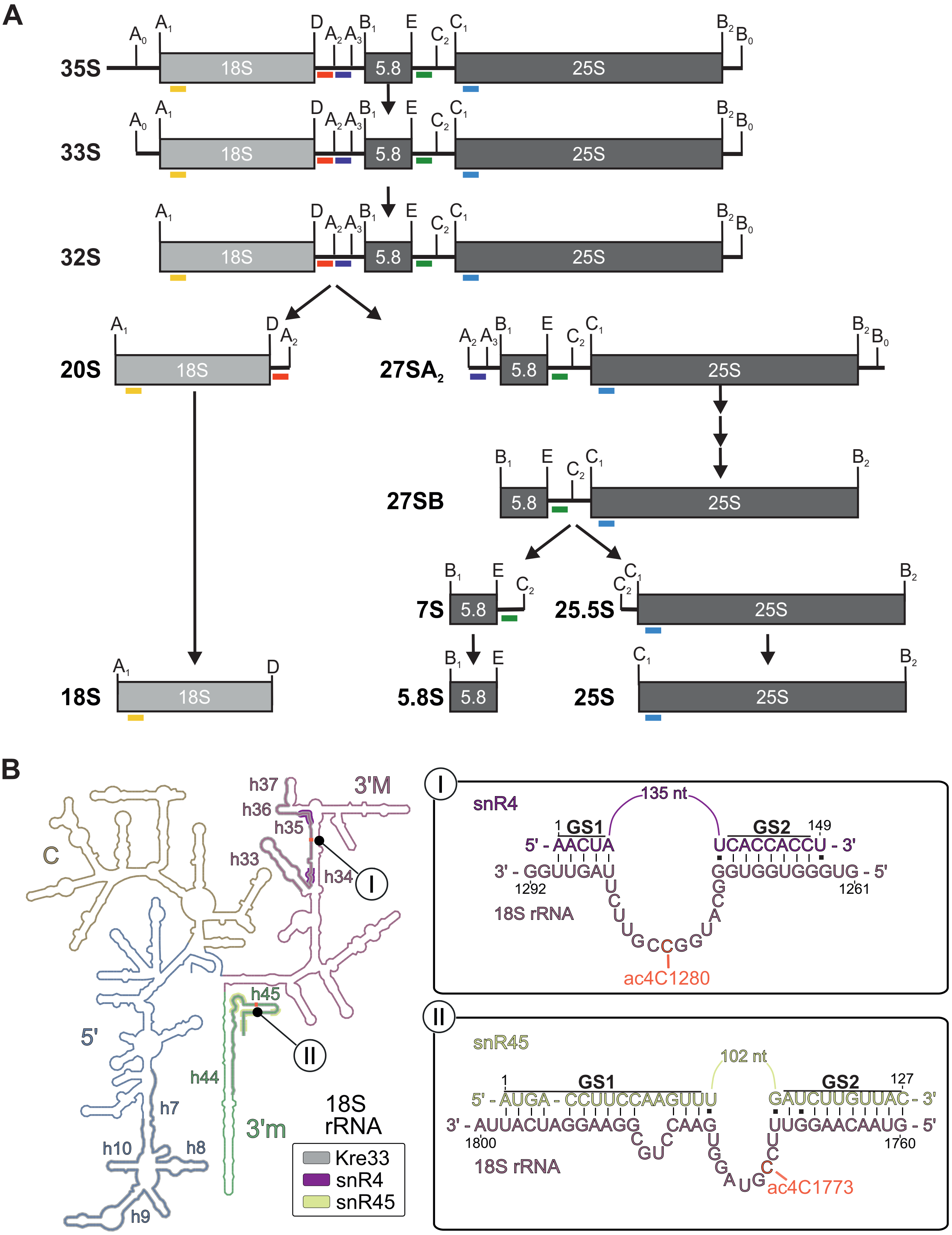

Supplementary Figure S1: rRNA processing and binding of acetylation guide snoRNAs to rRNA. (A) Simplified overview of the yeast pre-rRNA processing pathway. The 35S pre-rRNA, containing the mature 18S, 5.8S and 25S rRNA, is displayed on top, and all pre-rRNA cleavage sites are indicated. Binding sites of probes used for the northern blot experiment in Supplementary Figure S6A are indicated by colored short lines, with the following coloring: 18S (yellow), 25S (blue), Da_2 (red), A_2A_3 (purple), EC_2 (green). (B) 18S rRNA secondary structure with snR4 and snR45 binding sites indicated. The zoom-ins show the predicted base-pairs of snR4 and snR45 with the 18S rRNA (1).

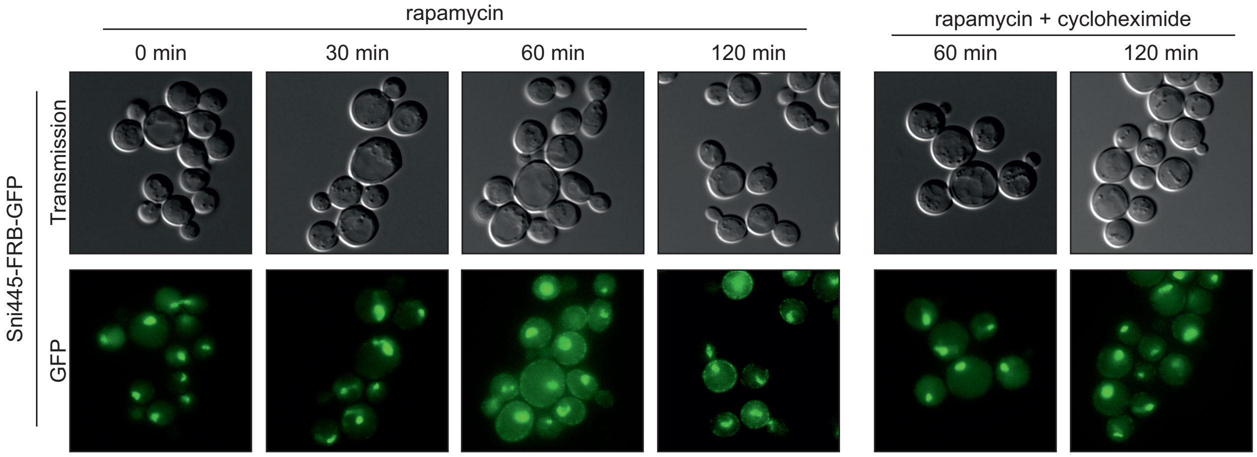

Supplementary Figure S2: Sni445 localizes exclusively to the nucleus. Anchor-away yeast reporter cells (2) expressing Sni445-FRB-GFP were treated during logarithmic growth phase with 1 µg/ml rapamycin or with 1 µg/ml rapamycin plus 10 µg/ml cycloheximide (to inhibit de-novo protein synthesis), followed by fluorescence microscopy. Samples were collected after 30, 60, and 120 min of rapamycin treatment, or after 60 and 120 min of combined rapamycin and cycloheximide treatment.

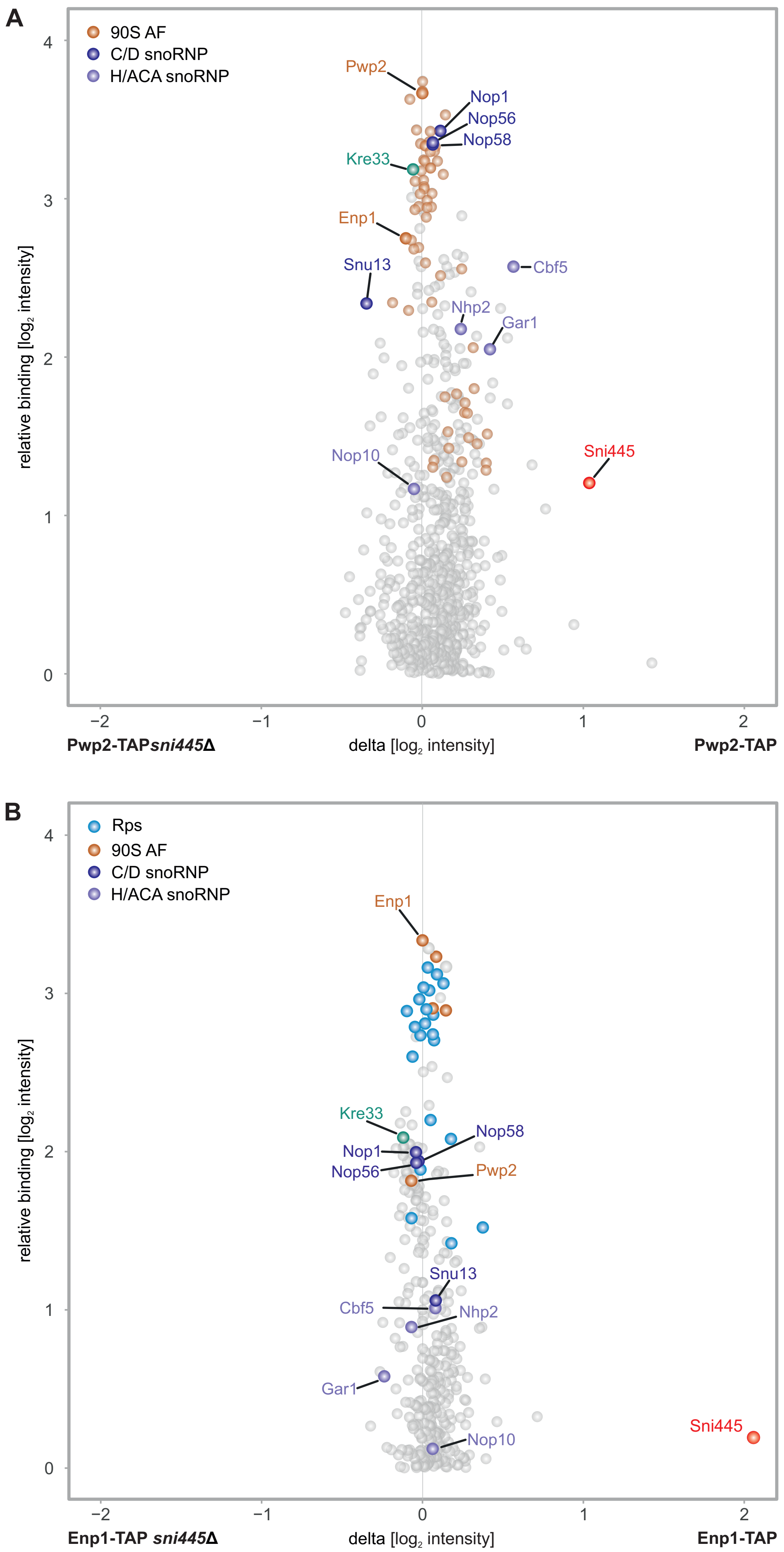

Supplementary Figure S3: **Sni445 is co-purified with 90S particles**. Pwp2-TAP (A) or Enp1-TAP (B) eluates from *SNI445* and *sni445*Δ strains were analyzed by label-free mass spectrometry in three biological replicates. z-score-normalized label-free quantification (LFQ) values were log$_2$-transformed and visualized. X-axis (delta [log$_2$ intensity]), difference of averaged log$_2$ intensity values of (A) ([Pwp2-TAP]-[Pwp2-TAP *sni445*Δ]) or (B) ([Enp1-TAP]-[Enp1-TAP *sni445*Δ]). Y-axis (relative binding [log$_2$ intensity]): averaged median-normalized label-free quantification from all experimental replicates. Only specifically bound proteins with values >0 (i.e. higher than the median) are shown. Proteins are annotated through color labels: Sni445 (red); box C/D proteins (blue); box H/ACA proteins (purple); 90S assembly factors (90S AF; brick); small subunit r-proteins (Rps; cyan).

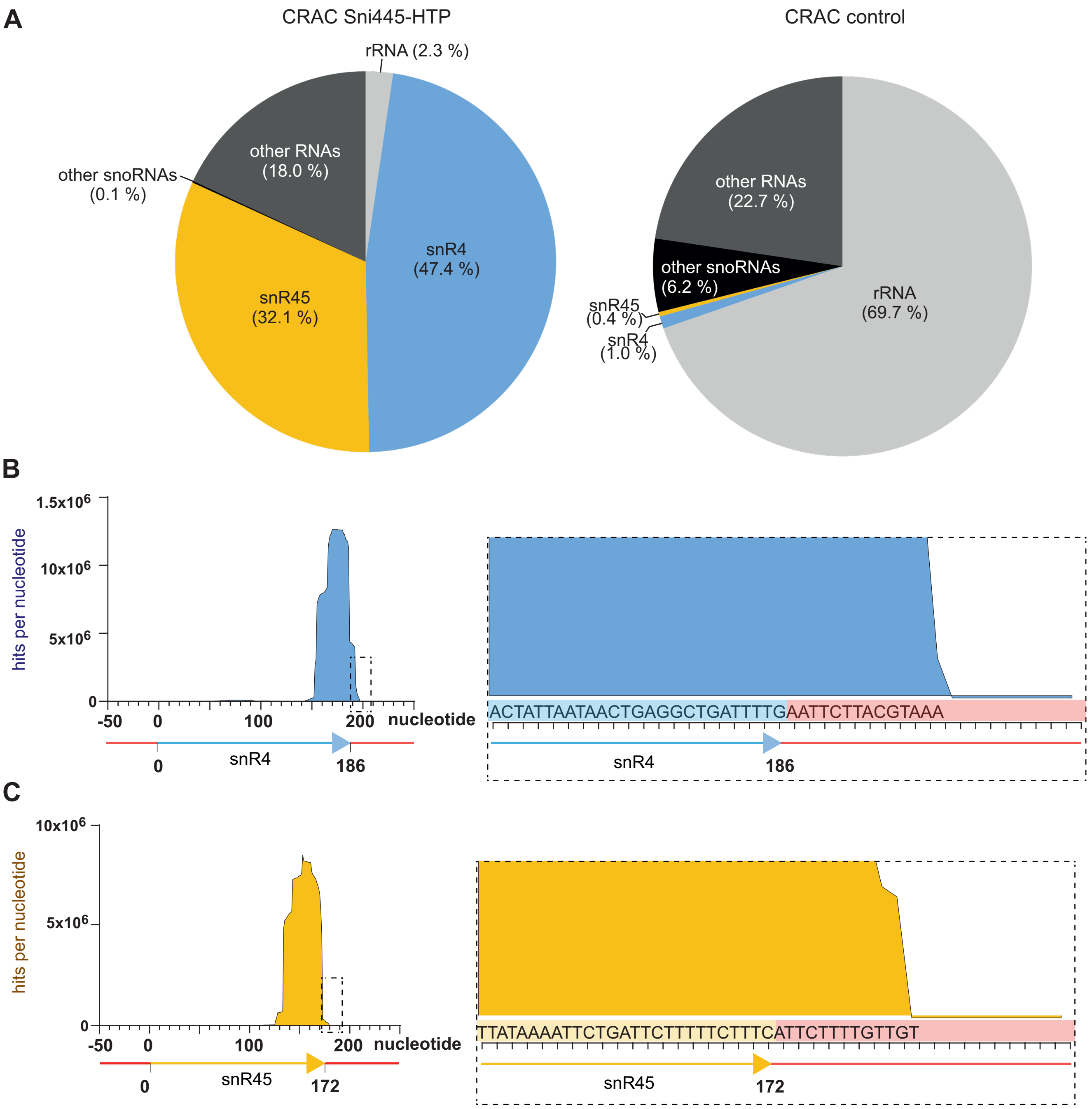

Supplementary Figure S4: Sni445 CRAC analysis. (A) Pie charts showing the distribution of RNA classes identified in CRAC experiments, comparing Sni445-HTP results from Figure 2A (left panel) to an untagged negative control strain (right panel). (B, C) Analysis of CRAC reads mapping to snR4 and snR45, including 50 nt upstream and downstream of the genes, reveals that a subset of crosslinked snoRNAs carry ~10 nucleotide extensions at their 3' ends. The right panels show zoom-ins of the 3' regions; sequences highlighted in red correspond to 3' extensions absent from the mature snoRNAs.

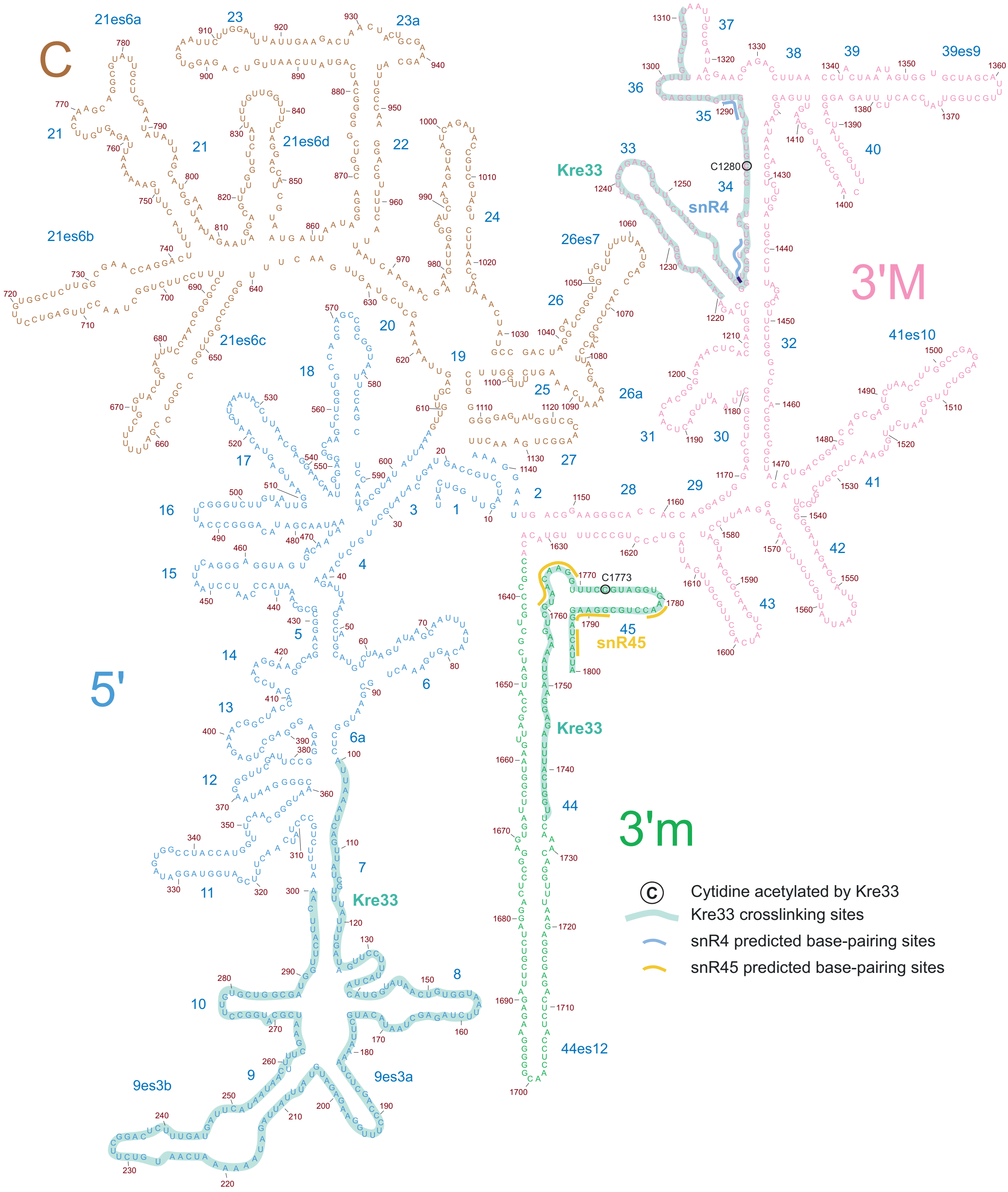

Supplementary Figure S5: Secondary structure of 18S rRNA. The four secondary structure domains, 5' domain (5'; blue), central domain (C; brown), 3' major domain (3'M; pink), and 3' minor domain (3'm; green) are highlighted. Base-pairing sites of snR4 (blue) and snR45 (yellow), as well as crosslinking sites of Kre33 (turquoise) are highlighted (1). Cytidines acetylated by Kre33 are indicated by circles.

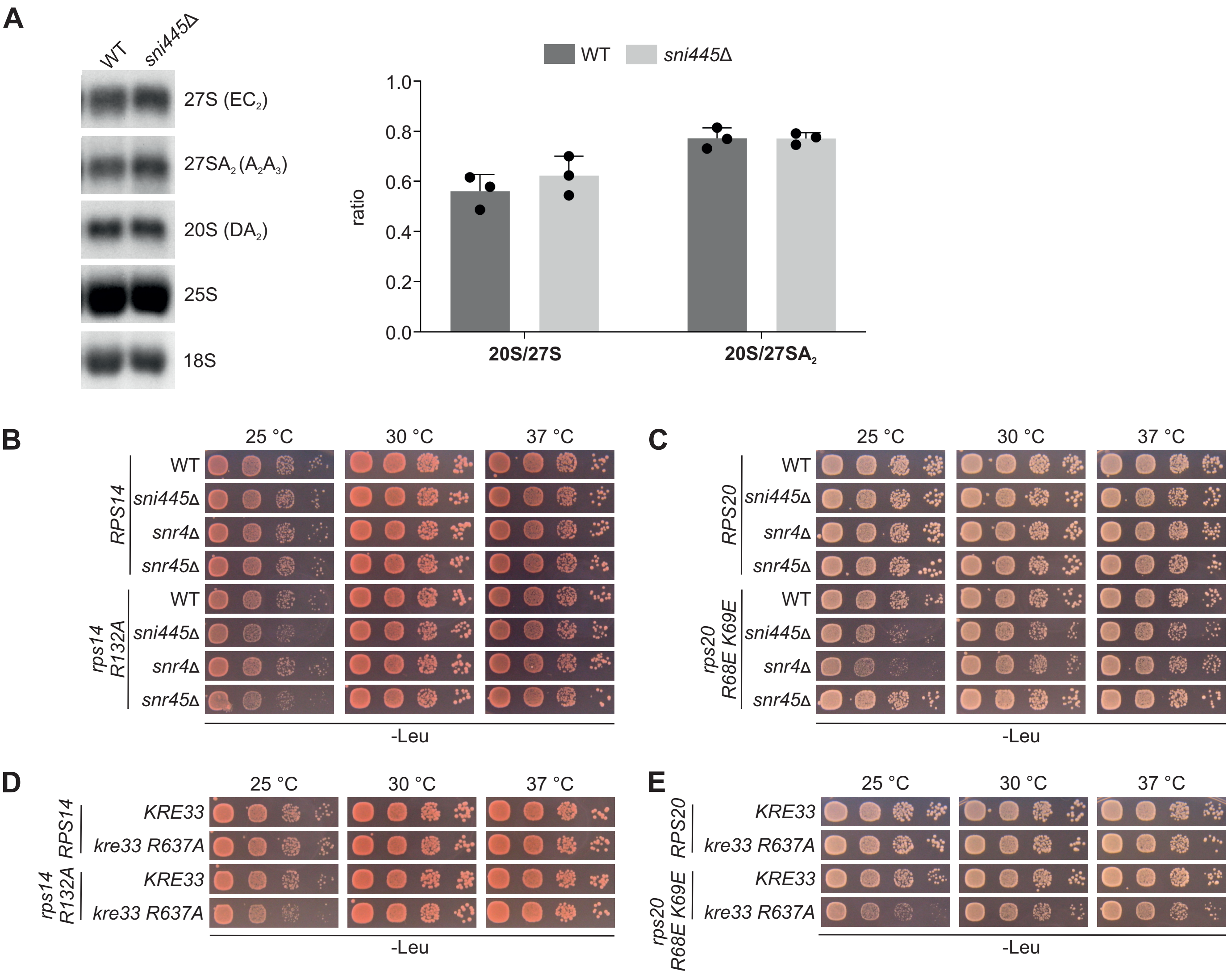

Supplementary Figure S6: Genetic interactions of *snr4* and *snr45* mutants are phenocopied by *sni445* and *kre33* mutants. (A) *sni445*Δ strains do not display detectable rRNA processing defects. RNA was extracted from *SNI445* wildtype (WT) or *sni445*Δ cells additionally carrying an *ENP1*-TAP fusion. Precursors of the large ribosomal subunit rRNA (27S, comprising the combined signal of 27SA$_2$, 27SA$_3$ and 27SB species, as well as 27SA$_2$), a precursor of the small subunit rRNA (20S), as well as mature 25S and 18S rRNA were detected. Probe binding sites are indicated in Supplementary Figure S1A. Right panel: quantification of three biological replicates shows that the ratio of pre-rRNAs in 40S precursors (20S) relative to 60S precursors (27S and 27SA$_2$) is not significantly altered upon deletion of *SNI445*. (B-E) Genetic interactions of *SNR4*, *SNR45*, *SNI445*, or *KRE33* with *rps14* or *rps20* mutants. The same genetic interactions as shown in Figures 3C, 3D, 4D, and 4E are presented; in addition to 25°C as in Figures 3 and 4, also growth at 30°C and 37°C is shown.

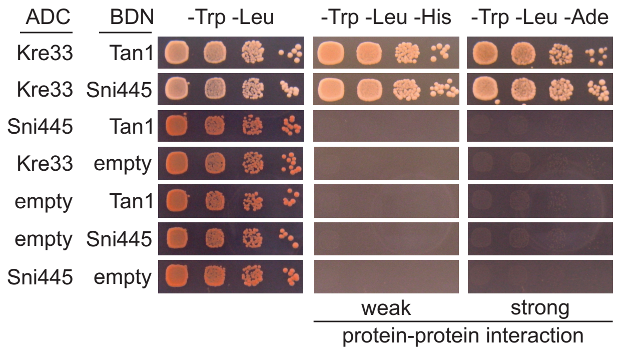

Supplementary Figure S7: Y2H interaction between Kre33 and Tan1. In addition to the combinations shown in Figure 4B, empty vector controls expressing the non-fused ADC or BDN are included. Growth on -Trp -Leu -His or -Trp -Leu -Ade plates, indicative of a Y2H interaction, is observed only when Kre33-ADC fusion protein is co-expressed with the BDN-Tan1 or the BDN-Sni445 fusion protein, but not when either interaction partner is expressed alone.

Supplementary Table S1. Yeast strains used in this study

name	genotype	source
W303	*ade2 leu2 his3 trp1 ura3*	(3)
W303 *ade3Δ*	*MAT*α *ade2 leu2 his3 trp1 ura3 ade3*Δ::kanMX4	(4)
W303 *ade3Δ*	*MAT*α *ade2 leu2 his3 trp1 ura3 ade3*Δ::natNT2	This study
Y2H PJ69-4A	*MAT***a** *trp1-901 leu2-3,112 ura3-52 his3-200 gal4Δ gal80Δ LYS2::GAL1-HIS3 GAL2-ADE2 met2::GAL7*-lacZ	(5)
Sni445-GFP Nop58-RedStar2	W303 *MAT***a** *NOP58*-RedStar2*::natNT2, SNI445*-GFP*::*HIS3MX6	This study
Sni445 -FRB-GFP	*MAT*α *leu2 ura3 his3 ade2 tor1-1 fpr1::*natNT2 *PMA1*-2xFKBP12*::TRP1 SNI445*-FRB-GFP*::*kanMX	This study
BY4741	*MAT***a** *his3 leu2 met15 ura3*	Euroscarf
Sni445-HTP	BY4741 *MAT***a** *SNI445-HTP::*klURA3	This study
Sni445-TAP	W303 *MAT***a** *SNI445*-TAP*::*HIS3MX6	This study
Sni445-FLAG	W303 *MAT*α *SNI445*-FLAG*::*natNT2	This study
Pwp2-TAP	W303 *MAT***a** *PWP2*-TAP*::*HIS3MX6	This study
Pwp2-TAP *sni445Δ*	W303 *MAT***a** *PWP2*-TAP*::*HIS3MX6 *sni445*Δ*::*kanMX	This study
Enp1-TAP	W303 *MAT***a** *ENP1*-TAP*::*HIS3MX6	(6)
Enp1-TAP *sni445Δ*	W303 *MAT***a** *ENP1*-TAP*::*HIS3MX6 *sni445*Δ*::*kanMX4	This study
RPS20-Shuffle	W303 *MAT*α *rps20*Δ*::*HIS3MX4 *ade3*Δ*::*kanMX4 [YCplac33-*RPS20*]	This study
RPS20-Shuffle *sni445Δ*	W303 *MAT*α *rps20*Δ*::*HIS3MX4 *ade3*Δ*::*kanMX4 [YCplac33-*RPS20*] *sni445*Δ*::*natNT2	This study
RPS20-Shuffle *snr4Δ*	W303 *MAT*α *rps20*Δ*::*HIS3MX4 *ade3*Δ*::*kanMX4 [YCplac33-*RPS20*] *snr4*Δ*::*natNT2	This study
RPS20-Shuffle *snr45Δ*	W303 *MAT*α *rps20*Δ*::*HIS3MX4 *ade3*Δ*::*kanMX4 [YCplac33-*RPS20*] *snr45*Δ*::*natNT2	This study
RPS14-Shuffle	W303 *MAT***a** *rps14a*Δ*::*HIS3MX6 *rps14b*Δ*::*natNT2 [YCplac33-*RPS14A*]	This study
RPS14-Shuffle *sni445Δ*	W303 *MAT***a** *rps14a*Δ*::*HIS3MX6 *rps14b*Δ*::*natNT2 *sni445*Δ*::*kanMX4 [YCplac33-*RPS14A*]	This study

RPS14-Shuffle *snr4*Δ	W303 *MAT***a** *rps14a*Δ*::*HIS3MX6 *rps14b*Δ*::*natNT2 *snr4*Δ*::*kanMX4 [YCplac33-*RPS14A*]	This study
RPS14-Shuffle *snr45*Δ	W303 *MAT***a** *rps14a*Δ*::*HIS3MX6 *rps14b*Δ*::*natNT2 *sni45*Δ*::*kanMX4 [YCplac33-*RPS14A*]	This study
RPS14-Shuffle *KRE33* WT	W303 *MAT***a** *rps14a*Δ*::*HIS3MX6 *rps14b*Δ*::*natNT2 [YCplac33-*RPS14A*] *KRE33::*hphNT1	This study
RPS14-Shuffle *kre33.R637A*	W303 *MAT***a** *rps14a*Δ*::*HIS3MX6 *rps14b*Δ*::*natNT2 [YCplac33-*RPS14A*] *kre33.R637A::*hphNT1	This study
RPS20-Shuffle *KRE33 WT*	W303 *MAT*α *rps20*Δ*::*HIS3MX4 *ade3*Δ*::*kanMX4 [YCplac33-*RPS20] KRE33::*hphNT1	This study
RPS20-Shuffle *kre33.R637A*	W303 *MAT*α *rps20*Δ*::*HIS3MX4 *ade3::*ΔkanMX4 [YCplac33-*RPS20*] *kre33.R637A::*hphNT1	This study
*sni445*Δ	W303 *MAT*α *sni445*Δ:: klTRP1	This study
*snr4*Δ	W303 *MAT*α *snr4*Δ::kanMX6	This study
*snr45*Δ	W303 *MAT*α *snr45*Δ:: hphNT1	This study
Sni445-TAP Nop58-FLAG	W303 *MAT*α *SNI445*-TAP::klURA3 *NOP58*-FLAG::natNT2	This study

Supplementary Table S2. Plasmids used in this study

**name**	**genotype**	**source**
pFA6a-HIS3MX4	for chromosomal deletion	(8)
pFA6a-kanMX4	for chromosomal deletion	(8)
pFA6a-natNT2	for chromosomal deletion	(9)
pFA6a-klTRP1	for chromosomal deletion	(10)
pFA6a-hphNT1	for chromosomal deletion	(9)
pFA6a-*KRE33*(1800-Stop)-150 downstream of *KRE33* - hphNT1 - 226-682 downstream *KRE33*	for chromosomal insertion of *KRE33* wild-type sequence	This study
pFA6a- *kre33 R636A*(1800-Stop)-150 downstream of *KRE33* - hphNT1 - 226-682 downstream *KRE33*	for chromosomal insertion of *kre33 R636A* mutant sequence	This study
pFA6a-GFP(S65T)::HIS3MX4	for C-terminal tagging	(11)

pFA6a-TAP::HIS3MX4	for C-terminal tagging	(12)
pBS1539 HTP::klURA3	for C-terminal tagging	(13)
pFA6a-FRB-GFP::kanMX	for C-terminal FRB-GFP tagging	(2)
pFA6a Flag-TCYC1-natNT2	for C-terminal tagging	(14)
pCUP111-yEGFP-(GA)5-TurboID-2xHA (pDK9295)	*CEN, LEU2,* P*CUP1, yEGFP-*TurboID-2xHA, T*ADH1*	(15)
pCUP111-SV40NLS-yEGFP-(GA)5-TurboID-2xHA (pDK9296)	*CEN, LEU2,* P*CUP1, SV40NLS-*yEGFP-TurboID-2xHA, T*ADH1*	(16)
pCUP111-*SNI445*-(GA)5-TurboID-2xHA	*CEN, LEU2,* P*CUP1, SNI445-*TurboID-2xHA, T*ADH1*	This study
pG4BDN22-*SNI445*	CEN, *TRP1,* P*ADH1,* T*ADH1*, N-terminal G4BD-cMyc	This study
pGAG4ADC111-*KRE33*	CEN, *LEU2,* P*ADH1,* T*ADH1*, C-terminal $(GA)_5$-G4AD-HA	This study
pG4BDN22-*TAN1*	CEN, *TRP1,* P*ADH1,* T*ADH1*, N-terminal G4BD-cMyc	This study
YCplac111-*RPS20*	*CEN*, *LEU2*, *RPS20*	(17)
YCplac111-*rps20.R68E/K69E*	*CEN*, *LEU2*, *rps20 R68/K69>E*	(17)
YCplac111-*RPS14A*	*CEN*, *LEU2*, *RPS14A*	(18)
YCplac111-*rps14a.R132A*	*CEN*, *LEU2*, *rps14a.R132A*	This study
YCplac111-*rps14a.R136A*	*CEN*, *LEU2*, *rps14a.R136A*	(18)

Unless otherwise stated, all genes were cloned with their natural promoters.

Supplementary Table S3: Sni445 TurboID-based proximity labeling data.

Supplementary References

1. Sharma, S., Yang, J., van Nues, R., Watzinger, P., Kötter, P., Lafontaine, D.L.J., Granneman, S. and Entian, K.-D. (2017) Specialized box C/D snoRNPs act as antisense guides to target RNA base acetylation, *PLoS Genet.,* **13,** e1006804.
2. Haruki, H., Nishikawa, J. and Laemmli, U.K. (2008) The anchor-away technique: rapid, conditional establishment of yeast mutant phenotypes, *Mol Cell.,* **31,** 925–932.
3. Thomas, B.J. and Rothstein, R. (1989) Elevated recombination rates in transcriptionally active DNA, *Cell,* **56,** 619–630.
4. Kressler, D., Doère, M., Rojo, M. and Linder, P. (1999) Synthetic lethality with conditional *dbp6* alleles identifies Rsa1p, a nucleoplasmic protein involved in the assembly of 60S ribosomal subunits, *Mol Cell Biol.,* **19,** 8633–8645.
5. James, P., Halladay, J. and Craig, E.A. (1996) Genomic libraries and a host strain designed for highly efficient two-hybrid selection in yeast, *Genetics,* **144,** 1425–1436.
6. Mitterer, V., Murat, G., Réty, S., Blaud, M., Delbos, L., Stanborough, T., Bergler, H., Leulliot, N., Kressler, D. and Pertschy, B. (2016) Sequential domain assembly of ribosomal protein S3 drives 40S subunit maturation, *Nat Commun.,* **7,** 10336.
7. Rössler, I., Embacher, J., Pillet, B., Murat, G., Liesinger, L., Hafner, J., Unterluggauer, J.J., Birner-Gruenberger, R., Kressler, D. and Pertschy, B. (2019) Tsr4 and Nap1, two novel members of the ribosomal protein chaperOME, *Nucleic Acids Res.,* **47,** 6984–7002.
8. Wach, A., Brachat, A., Alberti-Segui, C., Rebischung, C. and Philippsen, P. (1997) Heterologous HIS3 marker and GFP reporter modules for PCR-targeting in Saccharomyces cerevisiae, *Yeast,* **13,** 1065–1075.
9. Janke, C., Magiera, M.M., Rathfelder, N., Taxis, C., Reber, S., Maekawa, H., Moreno-Borchart, A., Doenges, G., Schwob, E. and Schiebel, E. *et al.* (2004) A versatile toolbox for PCR-based tagging of yeast genes: new fluorescent proteins, more markers and promoter substitution cassettes, *Yeast,* **21,** 947–962.
10. Pillet, B., Méndez-Godoy, A., Murat, G., Favre, S., Stumpe, M., Falquet, L. and Kressler, D. (2022) Dedicated chaperones coordinate co-translational regulation of ribosomal protein production with ribosome assembly to preserve proteostasis, *Elife,* **11.**
11. Longtine, M.S., McKenzie, A., Demarini, D.J., Shah, N.G., Wach, A., Brachat, A., Philippsen, P. and Pringle, J.R. (1998) Additional modules for versatile and economical PCR-based gene deletion and modification in Saccharomyces cerevisiae, *Yeast,* **14,** 953–961.

12. Pausch, P., Singh, U., Ahmed, Y.L., Pillet, B., Murat, G., Altegoer, F., Stier, G., Thoms, M., Hurt, E. and Sinning, I. *et al.* (2015) Co-translational capturing of nascent ribosomal proteins by their dedicated chaperones, *Nat Commun.,* **6,** 7494.

13. Granneman, S., Kudla, G., Petfalski, E. and Tollervey, D. (2009) Identification of protein binding sites on U3 snoRNA and pre-rRNA by UV cross-linking and high-throughput analysis of cDNAs, *Proceedings of the National Academy of Sciences of the United States of America,* **106,** 9613–9618.

14. Kater, L., Thoms, M., Barrio-Garcia, C., Cheng, J., Ismail, S., Ahmed, Y.L., Bange, G., Kressler, D., Berninghausen, O. and Sinning, I. *et al.* (2017) Visualizing the Assembly Pathway of Nucleolar Pre-60S Ribosomes, *Cell,* **171,** 1599-1610.e14.

15. Steiner, A., Favre, S., Mack, M., Hausharter, A., Pillet, B., Hafner, J., Mitterer, V., Kressler, D., Pertschy, B. and Zierler, I. (2023) Dissecting the Nuclear Import of the Ribosomal Protein Rps2 (uS5), *Biomolecules,* **13.**

16. Bhutada, P., Favre, S., Jaafar, M., Hafner, J., Liesinger, L., Unterweger, S., Bischof, K., Darnhofer, B., Siva Sankar, D. and Rechberger, G. *et al.* (2022) Rbp95 binds to 25S rRNA helix H95 and cooperates with the Npa1 complex during early pre-60S particle maturation, *Nucleic Acids Res.,* **50,** 10053–10077.

17. Mitterer, V., Shayan, R., Ferreira-Cerca, S., Murat, G., Enne, T., Rinaldi, D., Weigl, S., Omanic, H., Gleizes, P.-E. and Kressler, D. *et al.* (2019) Conformational proofreading of distant 40S ribosomal subunit maturation events by a long-range communication mechanism, *Nat Commun.,* **10,** 2754.

18. García-Gómez, J.J., Fernández-Pevida, A., Lebaron, S., Rosado, I.V., Tollervey, D., Kressler, D. and de la Cruz, J. (2014) Final pre-40S maturation depends on the functional integrity of the 60S subunit ribosomal protein L3, *PLoS Genet.,* **10,** e1004205.
